# Evaluation of arthroscopy and macroscopic scoring

**DOI:** 10.1186/ar2714

**Published:** 2009-06-02

**Authors:** Erik af Klint, Anca I Catrina, Peter Matt, Petra Neregråd, Jon Lampa, Ann-Kristin Ulfgren, Lars Klareskog, Staffan Lindblad

**Affiliations:** 1 Rheumatology Unit, Department of Medicine, Karolinska Institutet and Karolinska University Hospital, Building D2:01, S-171 76 Stockholm, Sweden

## Abstract

**Introduction:**

Arthroscopy is a minimally invasive technique for retrieving synovial biopsies in rheumatology during the past 20 years. Vital for its use is continual evaluation of its safety and efficacy. Important for sampling is the fact of intraarticular variation for synovial markers. For microscopic measurements scoring systems have been developed and validated, but for macroscopic evaluations there is a need for further comprehensive description and validation of equivalent scoring systems.

**Methods:**

We studied the complication rate and yield of arthroscopies performed at our clinic between 1998 and 2005. We also created and evaluated a macroscopic score set of instructions for synovitis.

**Results:**

Of 408 procedures, we had two major and one minor complication; two haemarthrosis and one wound infection, respectively. Pain was most often not a problem, but 12 procedures had to be prematurely ended due to pain. Yield of biopsies adequate for histology were 83% over all, 94% for knee joints and 34% for smaller joints. Video printer photographs of synovium taken during arthroscopy were jointly and individually reviewed by seven raters in several settings, and intra and inter rater variation was calculated. A macroscopic synovial scoring system for arthroscopy was created (Macro-score), based upon hypertrophy, vascularity and global synovitis. These written instructions were evaluated by five control-raters, and when evaluated individual parameters were without greater intra or inter rater variability, indicating that the score is reliable and easy to use.

**Conclusions:**

In our hands rheumatologic arthroscopy is a safe method with very few complications. For knee joints it is a reliable method to retrieve representative tissue in clinical longitudinal studies. We also created an easy to use macroscopic score, that needs to be validated against other methodologies. We hope it will be of value in further developing international standards in this area.

## Introduction

Diseases causing chronic inflammation in joints are common and often debilitating conditions. The synovial membrane (SM) is the primary target organ for the immune system in many chronic arthritides, and particularly in rheumatoid arthritis (RA) where a pannus of cells is formed, eroding cartilage and bone. Consequently, it is to be expected that investigations of the SM will provide clues to the pathogenesis of disease and effect of therapy. A number of studies have shown that the inflammatory changes in the synovium correlate with clinical [1-6], as well as radiological [7-10], outcomes.

Effects of different treatments [11-32] on these patterns have been studied and efforts have been made to investigate whether synovial histology markers could be used to evaluate the effect of a drug with some success [27,30,32]. Sampling of the synovial membrane has also been used as a 'proof of concept' prior to [33] or early on in clinical trials [34] of new drugs. Importantly, more recent studies have also found predictive markers of clinical effect [28,31,35]; however, more work needs to be conducted before we have simple markers enabling physicians to individually tailor medication. So far these markers are exclusively present in the synovium, the target organ of the inflammation, requiring surgical sampling of tissue.

Arthroscopy is a minimally invasive technique, traditionally used by orthopaedic surgeons, which has evolved as a research instrument in rheumatology to permit retrieval of SM during the past 20 years. Vital for its use is continual evaluation of its safety and efficacy, and the fact that there is intra-articular variation for synovial markers is important for sampling [36]. For microscopic measurements scoring systems have been developed and validated [37,38], but for macroscopic evaluations there is a need for further comprehensive description and validation of equivalent scoring systems.

In this report we aim to document our own experience with arthroscopy [36,39-51] describing the method, its safety and evaluating a macroscopic scoring system of synovitis developed by us.

## Materials and methods

### Patients

For seven years, from September 1999 to September 2005, 234 patients were recruited from the rheumatology clinic at the Karolinska University Hospital, and three patients from private rheumatologists. For research purposes, 210 patients were recruited, and 27 patients were recruited for clinical routine examination. Except for 10 healthy individuals, all patients had clinically active arthritis or joint pain at the time of arthroscopy. Indications for arthroscopy in clinical routine practice were mainly arthritis of unknown origin (mainly monoarthritis) or arthritis in singular joints without satisfying response to therapy. Projects for research purposes were primarily aimed at learning more about the early course of disease and the molecular mode of action of different anti-rheumatic treatments. Contraindications for arthroscopy were age below 18 years, prosthesis, clotting or bleeding deficiency, known allergy to local anaesthetics and cases where we were unable to communicate appropriately with the patient for psychological reasons or for language difficulties. Further, we did not include patients with septic arthritis, haemarthrosis, joint trauma or mechanical joint complications.

### Methods

All arthroscopies were performed in the same procedure room, designated for this and other small operative procedures requiring sterility and situated at our outpatient clinic. During most procedures three or more persons were involved; one or two operators (one teaching), one nurse and one assistant nurse (not in sterile dressing) taking care of tissue samples. Rod-lens arthroscopes (Karl Storz Gmbh, Tuttlingen, Germany) of three different dimensions (1.9 mm for proximal interphalangeal, metacarpophalangeal, wrist and elbow joints; 2.4 mm for shoulder, ankle and knee joints, and 4.0 mm for knee joints), all with a 30° angle, were used throughout. Spoon forceps (Karl Storz Gmbh, Tuttlingen, Germany) of different sizes were used to obtain the biopsies, the largest with a diameter of 3.5 mm, was used in knee joints. To minimise the effects of the procedure on the macroscopic appearance of the SM including circulation, no tourniquet was used, anaesthetic drug (xylocain) was used without adrenaline and maximum water pressure was put at 50 cm (for irrigation fluid). All arthroscopies were performed in accordance with the Helsinki Declaration, and where appropriate, ethical permission was given by the ethical committee at the Karolinska Institute, and written consent given by each patient before entering study.

Biopsies were put in cryotubes (Simport Plastics, Quebec, Canada) and frozen in precooled (-70°C) isopentane within two minutes (most often within one minute) after removal. They were stored until sectioned in -70°C. Before sectioning, biopsies were embedded in Tissue-Tek^® ^O.C.T. Compound (Sakura Finetek USA Inc, Torrance, California, USA). All biopsies were cut in -20°C (cryostat setting; 7 μm) and stained with H&E in a standard procedure. The sections were evaluated for adequate histology (inflammation and not subsynovial or fibrotic tissue) before further stainings were performed.

The arthroscopic procedure of the knee joint is detailed below, and is principally the same for other joints. The joint is examined from the outside for signs of inflammation (pain, swelling and hypertrophy of the joint capsule). Two entry portals are then localised (infralateral, supralateral and/or supramedial portals) and anaesthetised using 10 to 15 ml of xylocaine 10 mg/ml without adrenaline for skin and joint capsule. Disinfection and draping of the leg occurs. A minimal skin incision is made with a scalpel (<5 mm) for portals. The arthroscope portal and trocar are introduced and excessive synovial fluid is extracted and stored. The trocar is replaced by the arthroscope, and 10 ml of xylocaine is added in to the joint as it is filled with physiological saline for clear vision. The synovium is inspected systematically (suprapatellar pouch, lateral and medial recesses and gutters, leaving the tibiofemoral joint and posterior cavity of the joint). Water pressure is kept below 30 cm, so as not to interfere with vascularity. Biopsy sites are chosen according to maximal macroscopic inflammation and anatomical site (preferably three sites; one close to the cartilage-pannus-junction and two further away), and photographed.

**Figure 1:**
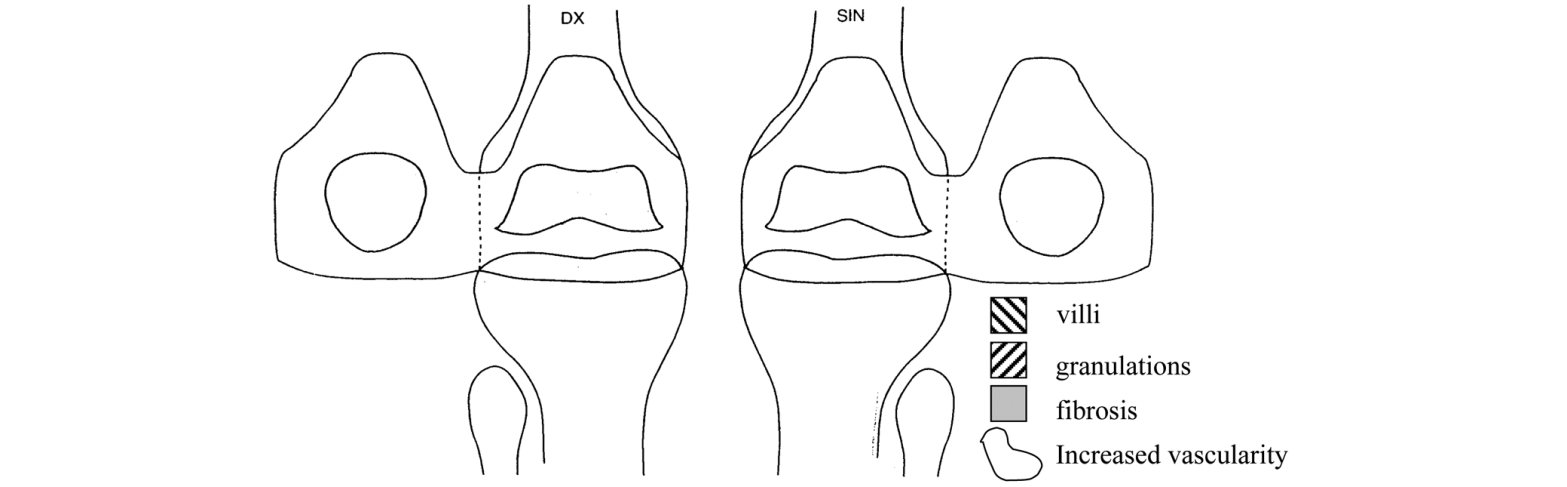
Arthroscopy map of the knee joints. The patella is folded sideways. Areas of hypertrophy (villi, granulations) are marked as indicated, areas of increased vascularity are encircled and biopsy sites are mapped immediately following each procedure by the performing arthroscopist. Dx, right side of patient; SIN, left side of patient.

The second portal for the biopsy forceps is then introduced. Synovial biopsies are retrieved, six to eight from each site, 12 to 24 per joint in total. Care is taken not to sample too deeply (subsynovial fat or capsule). Maximum time from biopsy sampling to freezing is set at two minutes, but is usually within 30 to 40 seconds. Joint lavage, to remove blood and debris, is used when needed for visuality throughout the procedure, but kept at a minimum so as not to interfere with treatment effect for drug evaluation (usually 300 to 600 ml, and never more than 1000 ml). Excessive fluid is extracted from the joint via both portals and intraarticular steroids are introduced via the arthroscope if indicated. Portals are removed, wounds cleaned, dried and taped with sterile strips then covered with sterile waterproof dressing. Immediately after the arthroscopy the operator maps the biopsy sites, as well as areas of increased vascularity, hypertrophy (granulations and villi) and fibrosis [36]. The map (Figure 1), together with the photographs taken, makes it possible to resample in close proximity to the primary site during consecutive procedures. The map and photos also allow retrospective analysis of biopsy sites and activity.

The entire procedure normally takes 40 to 60 minutes, and because only local anaesthetics and no sedation is used, the patient is able to walk immediately after the procedure. Before leaving the clinic, the patient is informed to avoid water contact on the wounds for two days. In order to quantitate possible complications and help patients after the procedure, the patient is contacted by the rheumatologist who performed the arthroscopy within the next few days. The occurrence of any complications is registered immediately after the arthroscopic procedure, at the following telephone contact and at consecutive contacts with the patients.

### Creation and evaluation of a macroscopic scoring system

Eight representative video printer photographs, taken at knee joint arthroscopy procedures, were chosen by one of the authors (EaK) to illustrate various features of macroscopic synovitis and serve as reference images. Scoring was made by EaK in relation to three parameters; hypertrophy, vascularity and synovitis. For each parameter a five point scale (0 to 4) was used.

Seven raters (rater 1 to rater 7) with different experience in arthroscopy were asked to score 50 different joint images that had been selected by EaK, after the scale had been described to each rater by EaK. The order of reading was randomised.

All scores were collected and inter-individual variation registered. Images that produced very variable scores were re-evaluated during a joint session. The eight reference image scores used were revised together. Ten new images were scored by all observers individually. These scores were compared, and a reference score was decided for each of these images.

From the experience gained in this study we prepared instructions for macroscopic scoring including characterised arthroscopic images, the 'Macro-score' [see Additional data files 1 and 2]. A new set of 50 images were scored by all raters at two occasions, with a minimum of 24 hours in between. The scores were analysed by descriptive analysis, including intra- and inter-rater variability and median scores.

Five control-raters with no previous experience of arthroscopy scored the same set of 50 images. They did not receive any further instructions than the Macro-score written instructions. Control-rater scores were analysed by descriptive statistics, including inter-rater variability and comparison to the median scores.

## Results

### Evaluation of the arthroscopic procedure for feasibility, complications and for yield of biopsies suitable for analysis

During seven years we performed 408 arthroscopic procedures in 237 patients (Tables 1 and 2 for patient characteristics). Tow out of three of the patients were classified as RA, fulfilling the American College of Rheumatology criteria [52]. The mean disease duration was 7.5 years (median 4 years) and 32% had disease duration less than one year. The mean arthritis duration was 43 weeks (median 12 weeks) and 72% were women. The maximum number of arthroscopies performed in one patient was four (six patients). The procedure itself typically lasted 40 to 60 minutes, with the most time required for preparation (disinfection, anaesthesia, draping etc). In a limited number of procedures (n = 38) only the operator and the assistant nurse were involved, showing the feasibility of this simplified approach.

**Table 1:** Patient characteristics at time of arthroscopy

Number of procedures	408
Number of patients	237
Age (mean)	52 years (range 20 to 85)
Sex	170 women/67 men
Disease duration (mean)	91 months (range 1 to 623)
Arthritis duration (mean)	43.5 weeks (range 0 to 564)
Patients on NSAIDs at arthroscopy	148
Patients on DMARDs at arthroscopy	99 (88 on methotrexate)

DMARDs = disease-modifying anti-rheumatic drugs; NSAIDs = non-steroidal anti-inflammatory drugs.

**Table 2:** Diagnosis at time of arthroscopy

Diagnosis	All patients (n)	RF+ (n)	Patients with early disease (<12 months) (n)	RF+ (n)
Rheumatoid arthritis	160	129	52	39
Undifferentiated monoarhtritis	13	4/11^a^	8^b^	2/6^a^
Undifferentiated oligoarthritis	9	2	4^b^	1
Undifferentiated polyarthritis	7	2	5^c^	1
Juvenile idiopathic arthritis	9	4	0	0
Adult Still's disease	1	0	0	0
Undifferentiated spondylarthritis	4	0	2	0
Psoriatic arthritis	12	0	2	0
Ankylosing spondylitis	1	0	0	0
Ulcerative colitis	2	0/1^d^	1	0
Undifferentiated systemic inflammatory disease with arthritis	1	0	0	0
Systemic lupus erythematosus	3	0	1	0
Cutaneous PAN with arthritis	1	0	0	0
Osteoarthritis	4	4	1	1
Healthy individuals	10	ND	NA	NA
Sum	237	141	76	42

All patients had clinically active inflammation or pain in at least the investigated joint (exceptions: healthy individuals). ^a ^Two patients were not tested for RF. ^b ^One RF+ patient later fulfilled RA classification criteria. ^c ^One RF-patient later fulfilled RA classification criteria, one RF-patient was later diagnosed positive for human parvovirus type B19 infection and one RF+ patient was later diagnosed with dermatomyositis. ^d ^One patient was not tested for RF.

NA = not applicable; ND = not determined; PAN = polyarteritis nodosa; RF = rheumatoid factor.

Except for two patients presenting with haemarthrosis three days and two weeks after the arthroscopy, respectively, no major complications were seen including deep vein thrombosis. A minor complication was one wound infection six weeks after arthroscopy at the supralateral portal where the patient had not removed the surgical strips. Intraarticular bleeding during the procedure could complicate biopsy sampling due to loss of vision, especially in small joints. However, in all of these cases bleeding stopped during or soon after the procedure. Pain in the investigated or other joint occurred in a small number of patients and in 12 (3%) cases pain restricted the procedure so that no or fewer biopsies were taken than were originally planned. In one patient pain was considered severe two weeks after the procedure. Additional medical treatment was received and the pain subsided slowly. In one patient the joint was not extending due to an inability to relax, requiring the arthroscopy to be terminated. In 18 procedures (11 knee joints and 7 small or medium-sized joints) we abstained from biopsies as no active synovitis could be visualised. In these cases synovium was either normal or fibrotic as assessed macroscopically. No patient had to stay at the hospital for more than one hour after the procedure, and no long-term complications were seen.

For knee arthroscopies, 97% of procedures were conducted as planned, and 95% of retrieved tissue was appropriate for histology, resulting in a total yield of 92% (Table 3). For smaller joints the quality of the biopsies was less consistent, resulting in 86% of the arthroscopies to be conducted as planned, with 40% of the retrieved tissue being appropriate for histology, resulting in a total yield of appropriate biopsy material of 34%. The major reason for this low yield in small joints was the difficulty in obtaining appropriate vision within the joint, which led to a practically blind biopsy in many cases.

**Table 3:** Approved histology per joint

Joint	Shoulder	Elbow	Wrist	MCP	PIP	Right knee	Left knee	Ankle	Sum
Procedures (n)	2	7	38	24	3	181	142	11	408
Biopsies approved by histology (%)	0	75	26	25	100	94	94	83	83

MCP = metacarpophalangeal; PIP = proximal interphalangeal.

### Creation and evaluation of a method for macroscopic scoring of synovitis during arthroscopy

**Figure 2:**
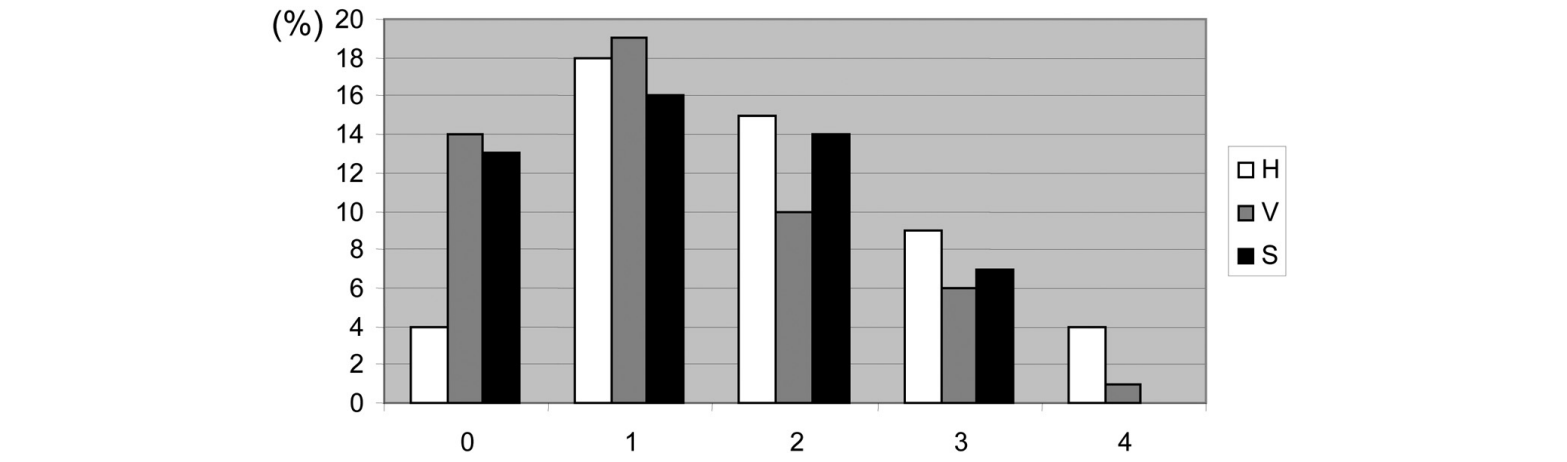
Median scores. Scores (%) sorted by median. Fifty images were scored for three parameters twice by seven raters, producing a total of 2100 scores. For S no images had a maximum median score of 4. H = hypertrophy; S = synovitis; V = vascularity.

We used printed photographs of synovitis obtained at arthroscopy in order to evaluate a scoring system that represents a development of a previous scoring procedure [36]. In order to quantitate how individual scorings may differ between different potential arthroscopists, we asked seven raters to assess 50 images. After following the steps outlined above, we analysed a second set of 50 images for intra- and inter-rater variation. Seven raters individually scored 50 images twice (with a minimum of 24 hours in between) for the three parameters, producing a total of 2100 individual scores (700 scores per parameter, 300 scores per rater and 14 scores per image and parameter). Median scores were calculated for all 50 images (Figure 2). Most images scored 1 and 2 in a five point scale (0 to 4, 0 representing no activity and 4 representing maximum activity) indicating low to medium levels of activity. No image rated a maximum median score of 4 for synovitis. More images had median scores of 0 for vascularity and synovitis compared with hypertrophy, indicating fibrotic inactive tissue. Some of these images and scores were used to create the written set of instructions called the Macro-score [see Additional data files 1 and 2].

### Intra-rater variation

**Figure 3:**
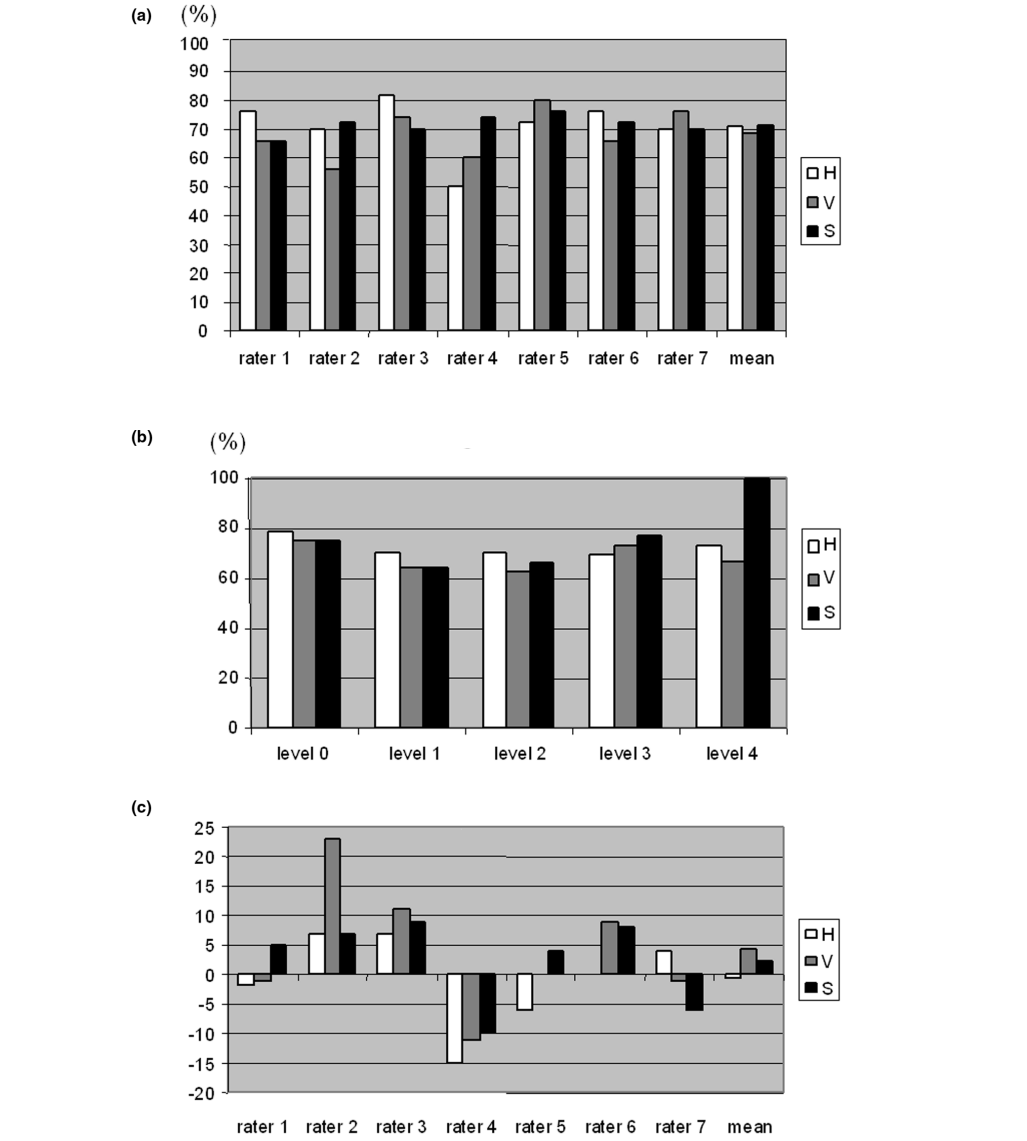
Intra-rater variation. **(a) **Percentage of perfect matches (score 1 = score 2) for individual raters. **(b) **Percentage of perfect matches (score 1 = score 2) at different scoring levels. **(c) **Rater consistency (sum of score 2 minus score 1). 0 indicates a high consistency between scoring sessions. A positive value indicates an increase in scores at the second scoring session. A negative value indicates a decrease in scores at the second scoring session.

Intra-rater variation was calculated from all 2100 scores. For hypertrophy 347 of 350 scores (99.1%) scored within one scoring step at the second rating session. For vascularity and synovitis the percentages were 98.9 and 99.1, respectively, showing a very low intra-individual variation. Each rater produced 150 scores, seven images yielded a scoring difference of more than one by one rater, the rest producing a maximum of one point in scoring difference. A mean perfect match (same score twice by the same rater) for hypertrophy was 71%, for vascularity 69% and for synovitis 71% (Figure 3a). When each parameter was analysed per score (Figure 3b), we could see that the best reproducibility was seen at the end points of each index. We could see a drift in consistency (Figure 3c), even though all raters calibrated themselves according to the jointly scored image set immediately before each scoring session.

### Inter-rater variation

**Figure 4:**
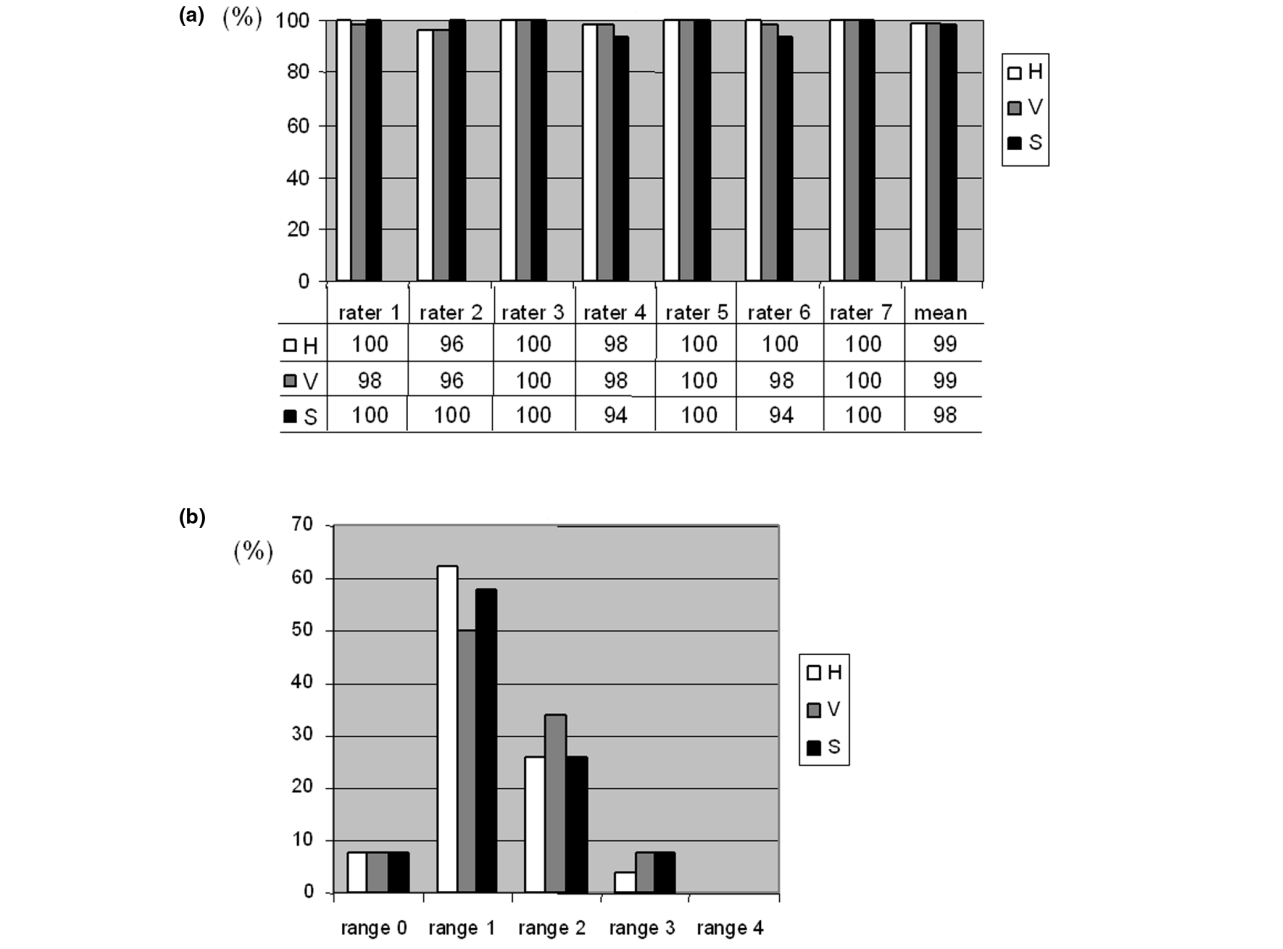
Inter-rater variation. **(a) **Distance to the median. Percentage of scores (from the first rating session) with an absolute distance from the median of one point or less. **(b) **Score range in percentage of total scores. A range of 0 is a perfect match of all seven scores for an image (seven raters). A range of one means that all scores are within two values. H = hypertrophy; S = synovitis; V = vascularity.

Inter-rater variation was calculated from the first set of scores by each rater, in all 1050 scores. Of 1050 individual scores, 1036 (98.7%) had an absolute deviation of one point or less from the median score. The deviation from the median varied between raters (Figure 4a); three raters averaged 100%, one rater averaged 99% and two raters averaged 97% of scores deviating by one point or less from the median, respectively, resulting in a mean of 99% of scores within one point of the median. We also analysed inter-rater variation by calculating the range of scores for each image parameter, given by the seven raters (Figure 4b). Range was two points or less in 139 (92.7%) of 150 image scores. This analysis shows that most scores were indeed close to one another and that raters were in close agreement (+/- one point) for almost all images.

### Comparison of control-rater scores to median scores of raters 1 to 7

**Figure 5:**
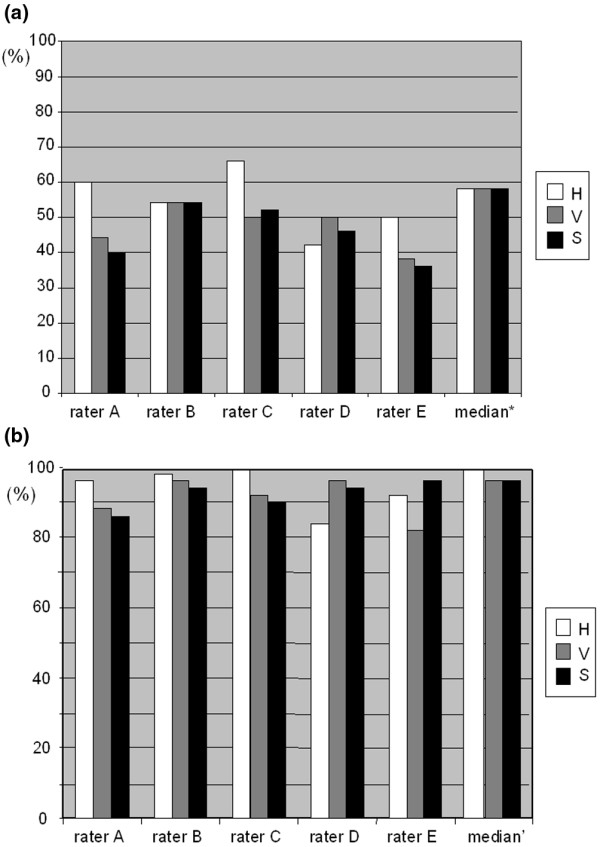
Control-rater variation (%). **(a) **Perfect matches (score of raters A to E = median score of raters 1 to 7). *Median score of raters A to E = median score of raters 1 to 7. **(b) **Distance to the median. Scores of raters A to E with an absolute distance from the median of raters 1 to 7 of one point or less (compare with Figure 4a). *Median scores of raters A to E with an absolute distance from the median of raters 1 to 7 of one point or less. H = hypertrophy; S = synovitis; V = vascularity.

The same set of 50 images that were scored by raters 1 to 7, were scored by five control-raters (rater A to E) not experienced in arthroscopy using the Macro-score [see Additional data files 1 and 2], producing 150 scores per rater, 650 scores in total. Average individual perfect matches (scores of control-raters equal to median scores of raters 1 to 7) varied between 41% and 56%. Perfect matches of median scores of raters A to E and 1 to 7 gave the best results (Figure 5a), 58% for each parameter, indicating that no individual parameter was harder to score. We also calculated percentage of scores with an absolute deviation of one point or less from the median score of raters 1 to 7. This turned out to be very high, between 82% and 100%. The average for each control-rater was between 90% and 96%. The median of the control-raters that were within one point of the median for raters 1 to 7 was even better; between 96% and 100%, giving an average of more than 97% of median scores within one point of the median by raters 1 to 7.

## Discussion

Arthroscopy in the hands of the orthopaedic surgeons is considered a safe and reliable method. In the surveys by Small [53] and Sherman and colleagues [54] the complication rate was between 2 and 5%. The feasibility and safety of arthroscopy in the hands of rheumatologists have been described in several reports over recent years; Kane and colleagues [55] identified 36 rheumatology centres in Europe, the USA and Australia that performed arthroscopy for clinical and research purposes. In this survey as well as in reports from individual centres (Table 4) few complications have been reported (1.5%). It is therefore comforting that complications seen in our series are at a low level (0.7%). Haemarthrosis was the most common major complication both from the survey (0.9%), as well as in our study (two patients or 0.5%). Importantly, other major complications, such as joint infection or deep vein thrombosis, did not occur at all in our study. In previous studies joint infections have been observed and been associated with irrigation volume [55], possibly as a marker of the length of the procedure. In our series, no procedure lasted for more than one hour, and we kept lavage volume at a minimum, which might thus contribute to lowering the risk of infection. The use of arthroscopy without tourniquet probably also reduces the risk of thrombosis or thrombophlebitis. In 111 procedures intraarticular steroids were administered in the examined joint at the end of the procedure, with no increased risk of adverse events.

**Table 4:** Complications of arthroscopy

**First author**	**AS (n)**	**Major (%)**	**Minor (%)**	**Haemarthrosis (%)**	**Joint infection (%)**
Sherman and colleagues [54]^‡^	2640	126 (4.8)	97 (3.7)	(2.0)	(<0.5)
Small [53]^‡^	10262	(1.7)		104 (1.0)	21 (0.2)
Szachnowski and colleagues [78]	335	(1.2)	(12.8)	nd	nd
Kuzmanova and colleagues [79]	206	4 (2.0)	15 (7.5)*	7 (3.5)	1 (0.5)
Reece and colleagues [80]	278	1 (0.4)	13 (4.7)	0	1
Wollaston and colleagues [81]	342	1 (0.3)	5 (1.5)	0	0
Smith and colleagues [56]	128	nd (studied pain in conjunction with AS)			
Baeten and colleagues [73]	150	0	(<10)	0	0
Gerlag and Tak [82]	>2000	<0.3			
Kane and colleagues [55]^☼^	16532	237 (1.5)		141 (0.9)	16 (0.1)
af Klint (present study)	408	2 (0.5)	1 (0.2)	2 (0.5)	0

^‡ ^Survey of orthopaedic AS. *Haemarthrosis was regarded as a minor complication. ^☼^Survey of AS.

AS = arthroscopy; nd = not determined.

Despite the use of local anaesthesia, pain may still be a problem. In a study of 50 patients [56] pain was reported in 50% during the procedure, and in 67% postoperatively. In that study, the best results for pain were seen in those who had femoral nerve blocks (most had no pain, and none had moderate or severe pain during or after the procedure) as opposed to local anaesthesia (none reported no pain during the procedure, severe pain was reported in 14% during and 10% after the procedure). Despite this only one patient declined to have a future second arthroscopy. In our study all received local anaesthesia. Occurrence of pain was notified in each case by the physician responsible for the procedure, but not captured in a formal protocol; we had to prematurely terminate the procedure in 3% of cases due to pain during the procedure. Only in one patient was there a severe pain that lasted for two weeks after the procedure. Also, the large majority of patients who were asked to a second arthroscopy consented to this procedure, indicating that the subjective experience of pain was not high. For future arthroscopic investigations, however, we advise that formal protocols for measurement of pain are introduced.

The yield of biopsies was highly satisfactory from the procedures directed towards the knee joints (94%) and acceptable from ankles joints (83%). The yield from small joints was, however, only 34%. This may be an obstacle that is possible to overcome as other groups have reported higher success rates for small joints including metacarpophalangeal joints [57]. As we were not able to reach this level of success, we draw the conclusion that sampling small joints requires special skills and training programs, and that new investigators should be aware of the difficulties. In our case, we decided to restrict the studies mainly to knees in studies where repeated biopsy sampling of the same joint was required [46-48,50,51].

In polyarticular arthritis patients a high degree of clinical interarticular variation of inflammation between different joints is clinically well recognised. This variation also occurs within the macroscopic and microscopic patterns of the synovium in the individual joint [36,39,58-64]. Some studies point to the correlation of macro and microscopic features [12,36,65-67], while others support that variation is limited [59,60,64,68,69]. To cope with this issue four main principles of sampling the synovium in large joints like the knee have been described: sampling predetermined sites [12]; sampling according to macroscopic signs of inflammation [36]; sampling predetermined sites and according to macroscopic signs of inflammation [65]; and sampling a limited number of predetermined sites to represent the whole joint [58,60,68]. Some studies point to the cartilage pannus junction [36,70,71] as the site of most active inflammation, although not in all aspects [72] implying the special significance of this region. Against this background, we have chosen to sample tissues based on maximum macroscopic inflammation far from and close to cartilage. When performing consecutive studies with repeated procedures, we prioritised sampling of the biopsies close to the same sites as the first. Samples from the same site are then compared for longitudinal outcome comparisons. To aid in this effort, we have made a simplified map of the knee joint (Figure 1) where macroscopic characteristics and biopsy sites are marked. Each site is also photographically documented by the arthroscope, now digitally stored as opposed to earlier video printer photographs.

There is an obvious need to correlate macroscopic findings and microscopic/molecular analysis of inflamed joints. Several different macroscopic scales have been used [36,65,73] and have been found to correlate with other SM features [12,36,65-67,74,75]. Notably few studies have been published on intra and inter-variation of different raters in macroscopic scoring. This issue was addressed by Reece and colleagues [76] using 44 video recordings of the arthroscopic procedure. Three observers reached good correlation for villous formations, the most pronounced form of hypertrophy, in RA; however, the results were not as good in spondylarthritis patients. Granulations and capillary hyperaemia were not reliable in this study. This is interesting as in the study by Lindblad and Hedfors [36], hyperaemia was the most important feature corresponding to microscopic changes, as also noted by oral surgeons [77]. Before reliable correlations can be made between macro and microscopic scoring systems these parameters need to be studied more extensively. This was one reason for undertaking this study.

In our study we chose not only to score global synovitis, but also to individually measure its main features; hypertrophy and vascularity. Hyperaemia was not included as an individual parameter due to its intrinsic variation physiologically as well as observationally. However, hyperaemia was included in the global synovitis score, in which all observed features of inflammation were included. Thus the score adds more information to the history of the synovitis as a global synovitis score of 0 is not always normal, but also includes previously active fibrotic changes scored as hypertrophy. This score set can be used for scoring entire joints where a video perhaps would be of greater value, but our aim was primarily to get low intra and inter-rater score variation in scoring biopsy sites for research purposes. In this study we report that intra-individual scoring variation was low; at the second scoring 99% of all scores were within one point of the first scores using a five-point scale. Further, a perfect match between first and second scoring sessions was reached in 70% of scores, and no single parameter had a substantially greater intrinsic variability. We also showed low inter-rater variation: 1036 of 1050 individual scores (98.7%) were within one point from the median score. The range was two points or less in 139 of 150 (92.7%) image scores. These numbers could presumably be improved with further training.

We also constructed an easy to use set of instructions for macroscopic evaluation, the Macro-score [see Additional data files 1 and 2]. These written instructions were tested on the same set of 50 photographs by five control-raters with no previous experience of arthroscopy. Without any other directions they scored well; between 41% and 56% of individual scores were equal to the median scores of the raters from the first group, and between 82% and 100% of scores were not more than one point from the median. The time to understand the score and score 50 images was about 2 to 2.5 hours, suggesting that the Macro-score is reliable and easy to use.

A weakness in this study was the lack of maximal inflammation scores. We presume that this is due to the population studied – we only included chronic polyarthritis patients, and no acute forms such as septic arthritis or gout. Given the clinical activity in these forms of arthritis, we would probably see more active synovitis in such images. At the other end of the spectrum we had a number of images depicting no inflammation, also from healthy individuals serving as controls. Interestingly, also healthy individuals with no history of joint problems could be seen to have minor inflammatory changes, in line with our earlier experience [41].

## Conclusions

It is our experience that rheumatological arthroscopy is a safe method with very few complications. For knee joints it is a reliable method to retrieve representative tissue in clinical longitudinal studies. We also created an easy to use macroscopic score that needs to be validated against other methodologies, which we hope will be of value in further developing international standards in this area.

## Abbreviations

H&E: haematoxylin and eosin; RA: rheumatoid arthritis; SM: synovial membrane.

## Competing interests

The authors declare that they have no competing interests.

## Authors' contributions

EaK participated in the design of the study, performed most arthroscopies, collected data from the arthroscopies, participated in designing the Macro-score, scored images, performed the statistical analysis and drafted the manuscript. AC performed arthroscopies, participated in designing the Macro-score, scored images and aided in the statistical analysis. PM, PN and JL performed arthroscopies and scored images. AU participated in the design of the study and scored images. LK participated in the design of the study and helped to draft the manuscript. SL participated in the design of the study, performed arthroscopies, participated in designing the Macro-score, scored images and helped to draft the manuscript. All authors read and approved the final manuscript.

## Supplementary Material

Additional file 1 A Word file containing a written set of instructions for scoring of synovitis by arthroscopy, images included, called the Macro-score. Click here for file

Additional file 2 A Powerpoint file containing calibrating images of synovitis for the Macro-score. Click here for file
