# Supplementary material for: Evaluation of arthroscopy and macroscopic scoring
Source: Arthritis Res Ther. 2009 Jun 2;11(3):R81. doi: 10.1186/ar2714 (PMC2714131; doi:10.1186/ar2714)
Supplement: Additional file 1 — A Word file containing a written set of instructions for scoring of synovitis by arthroscopy, images included, called the Macro-score. [file ar2714-S1.doc]

**MACRO-SCORE**

**Instructions for macroscopic scoring of synovitis by arthroscopy**

**Purpose of the score**

- To determine macroscopic synovitis activity of a biopsy/biopsy site/joint
- To be instrumental in choosing synovial membrane biopsy site

**Understanding the biology**

The synovial membrane (SM) is the inner membrane of the joint capsule surrounding a freely movable joint. It is loosely attached to the external fibrous capsule and secretes synovial fluid (SF). The SF is a clear, viscous fluid that is a dialysate of blood plasma but contains larger amounts of hyaluronic acid, as well as mucin, albumin, fat, and mineral salts. The SF serves two purposes; nutrition and lubrication of the cartilage. The SF is produced by the SM.

In the healthy individual, the SM consists of a lining layer (intima), which is only 1-2 cell layers thick. Beneath, is the sublining, or subintima, with supplying vessels, fat tissue and fibrous capsule. However, different parts of the joint have SM displaying different characteristics. In some areas the SM is not even present normally (e.g. cartilage, tendons). In other areas it is generally thicker and more hypertrophic and vascularized (e.g. the fat body of Hoffa) even under normal conditions.

The inflammatory process in the joint macroscopically follows the following scheme of events irrespective of the exact mechanism causing the synovitis:

1. Activation of the immune system without obvious macroscopic signs of change.
2. Cell infiltration and hypertrophy of the synovium seen as granulations and villi.
3. Ingrowth of activated vessels seen as increased vascularity and hyperemia.
4. When the synovitis is abolished only white fibrotic tissue remains, with no or few visible vessels.

**Macro-score – instructions**

Three complementary parameters have been chosen to grade macroscopic inflammatory activity:

- 1. **Hypertrophy:** This parameter grades visible synovial mass. The lowest score (0) represents a thin and transparent SM. Vessels are clearly visible, as is subsynovial tissue (e.g. fibrous capsule, fat or muscle). A score of 1 represents minor thickening, granulations score higher and villi give maximum score (4). The score does not take into account present or past inflammation activity; white fibrotic villi score the same as active hypervascularised villi.
  2. **Vascularity:** This parameter grades density of visible vessels. Thin and scattered vessels score 0. Subsequently fibrotic tissue, containing no or only few vessels, scores the same; 0. Increasing vascularity is graded by increasing scores. Densely packed vessels score 4, and intermediate in between. Hyperaemia is not included in this parameter.
  3. **Synovitis:** This parameter grades every visible aspect of synovitis as assessed globally by the observer. A higher score represents a higher degree of inflammation. White fibrotic tissue is scored 0, as is normal SM. Hyperaemia, which is not included in the vascularity index, is included here.

**General instructions:** The score is intended to grade synovitis in tissue biopsies. As the grade of inflammation may vary quite abruptly within a joint, this may be used to score the individual sample. It may also be used to score a biopsy site, or even the joint as a whole, taking areas of different appearances into account. **When scoring images the whole image should be scored as a mean, and not only the site with the most inflammation, unless otherwise specified.**

**Understanding macro-score:** Normal SM without any signs of activity scores 0/0/0 (hypertrophy/vascularity/synovitis). Thin, transparent villi (can be seen in a normal joint) score 1/0/0. Increased vascularity in still transparent SM without hypertrophy scores 0/1/1. Non-transparent, dense granulations with increased vascularity score 2-3/1-4/2-3. When the same features are seen in villi, they score higher; 3-4/1-4/3-4. The maximal score is 4/4/4. Each parameter thus gives different information contributing to the understanding of each image. A high hypertrophy index, combined with low vascularity and low synovitis indices, reflect a history of inflammation, now reduced. Fibrotic tissue scores 1-4/0/0. Observe that both normal and fibrotic tissue get a vascularity and synovitis score of 0. However, hypertrophy score will always be >1 for fibrosis. So, the different parameters complement each other.

**Learning macro-score:** We provide eight images, with their respective scores and comments, in order to understand the score. We also provide fifteen images that can be used as a practice. The key to each image of this set follows at the end.

We suggest that these images are used as a reference image library for the score.

**Using macro-score:** When we sample SM, we choose the most active sites, and avoid fibrotic tissue. We reason that both inflammatory changes and SM mass peak in these areas – thus being most suitable for tissue analysis, for purposes like assessing synovial inflammation over time in therapeutic studies, and for revealing pathogenic mechanisms in action at different time points in disease development. The score can thus be instrumental in deciding biopsy site or sites.

**Examples:**

**Score:** Hypertrophy/Vascularity/Synovitis

**Fig 1**

A normal knee joint, without any traces of previous episodes of inflammation. A thin transparent SM covers visible normal vessels. The proximal tibial cartilage is seen.

**Score:** 0/0/0

**Fig 2**

Low grade hypertrophy and increased vascularity form a low grade synovitis.

**Score:** 1/2/1

**Fig 3**

Granulations and few visible vessels form a medium grade synovitis.

**Score:** 3/1/2

**Fig 4**

Increased hypertrophy and high vascular density form a high grade synovitis.

**Score:** 2/3/3

**Fig 5**

Some hypertrophic changes and maximal density of vessels form a high grade synovitis, in a patient with resent onset (six weeks) rheumatoid arthritis (RA).

**Score:** 2/4/3

**Fig 6**

Fibrotic villi with no visible vessels form a zero grade synovitis, in a patient with longstanding RA.

**Score:** 3/0/0

**Note;** the hypertrophy index displays previous activity, distinguishing it from normal synovium, although no synovitis is presently active.

**Fig 7**

Maximal hypertrophy and much increased vascularity form maximal synovitis, in a patient with undifferentiated spondylarthritis.

**Score:** 4/3/4

**Fig 8**

Maximal inflammation of all parameters, in a patient with unclassified monoarthritis.

**Score:** 4/4/4

**Calibrating images:**

**Fig A**

**Fig B**

**Fig C**

**Fig D**

**Fig E**

**Fig F**

**Fig G**

**Fig H**

**Fig I**

**Fig J**

**Fig K**

**Fig L**

**Fig M**

**Fig N**

**Fig O**

**Key to figures A-O:**

**Hypertrophy/Vascularity/Synovitis**

**Fig A 3/3/3**

**Fig B 1/1/1**

**Fig C 3/3/3**

**Fig D 2/2/2**

**Fig E 0/0/0**

**Fig F 2/2/2**

**Fig G 1/1/1**

**Fig H 0/0/0**

**Fig I 2/0/0**

**Fig J 2/1/1**

**Fig K 4/4/4**

**Fig L 4/2/2**

**Fig M 2/4/2**

**Fig N 2/0/0**

**Fig O 3/2/2**
