# Supplementary material for: Evaluation of arthroscopy and macroscopic scoring
Source: Arthritis Res Ther. 2009 Jun 2;11(3):R81. doi: 10.1186/ar2714 (PMC2714131; doi:10.1186/ar2714)
Supplement: Additional file 2 — A Powerpoint file containing calibrating images of synovitis for the Macro-score. [file ar2714-S2.ppt]

## Slide 1
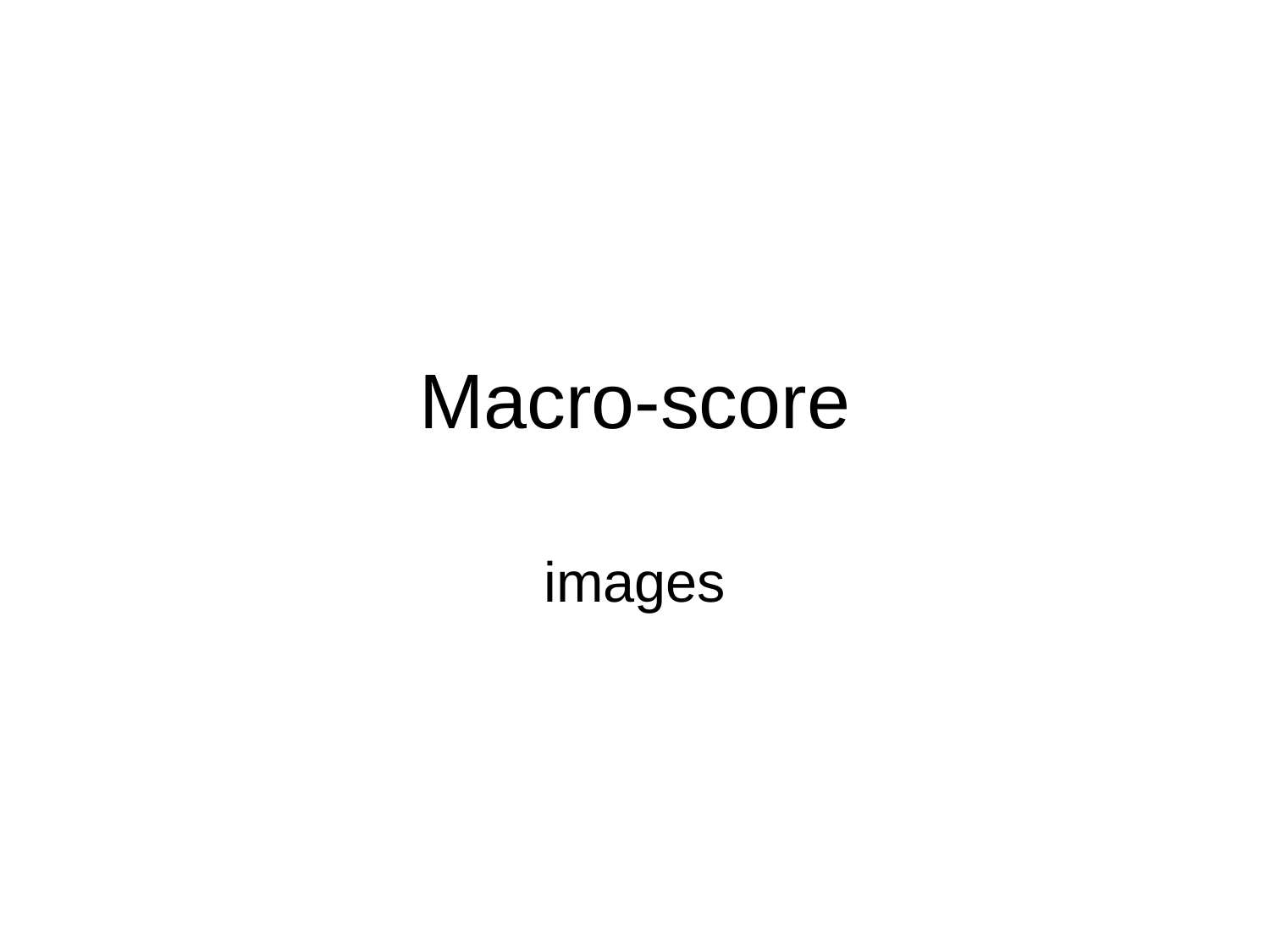

# Macro-score
images

## Slide 2
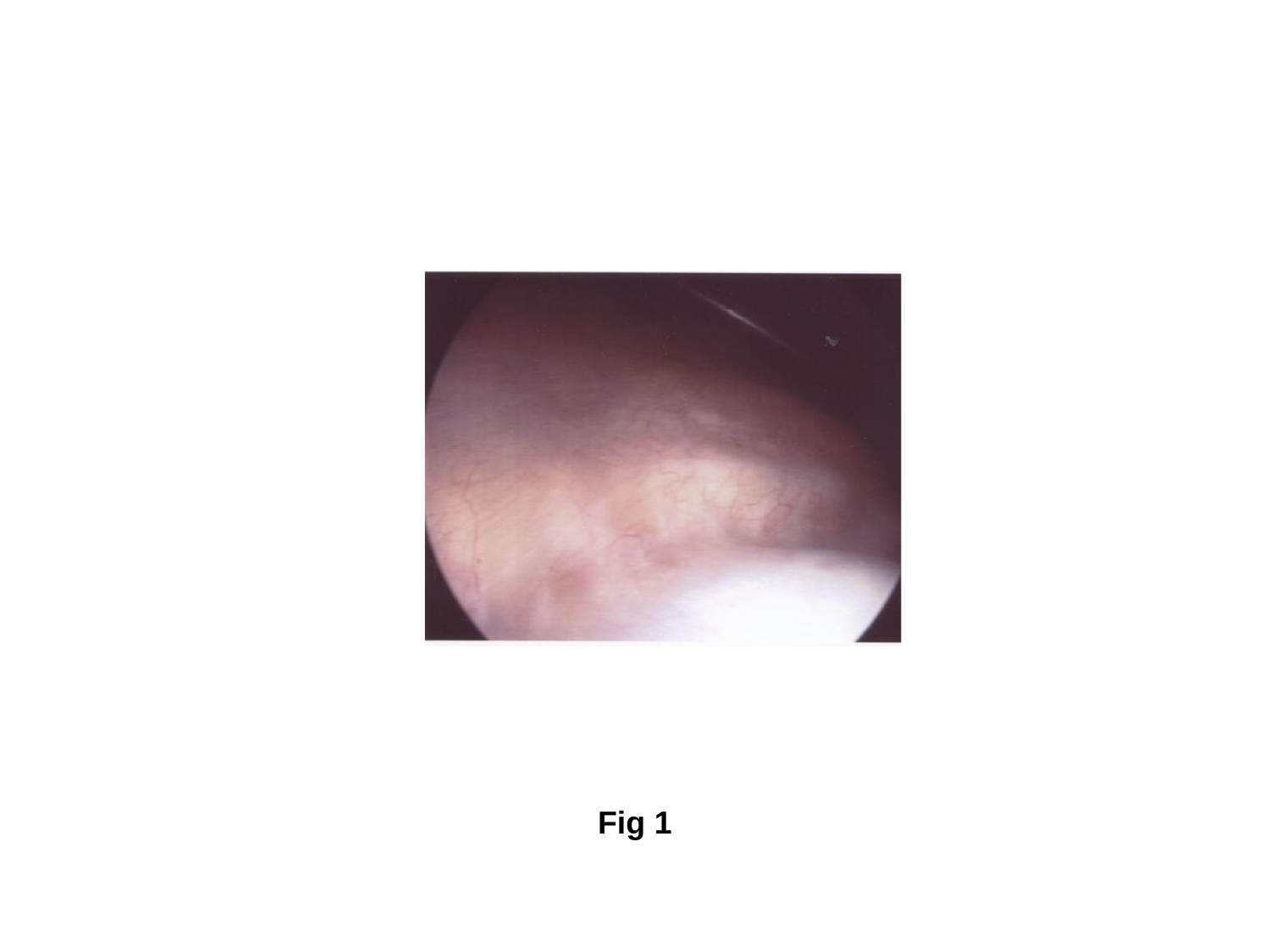

Fig 1

## Slide 3
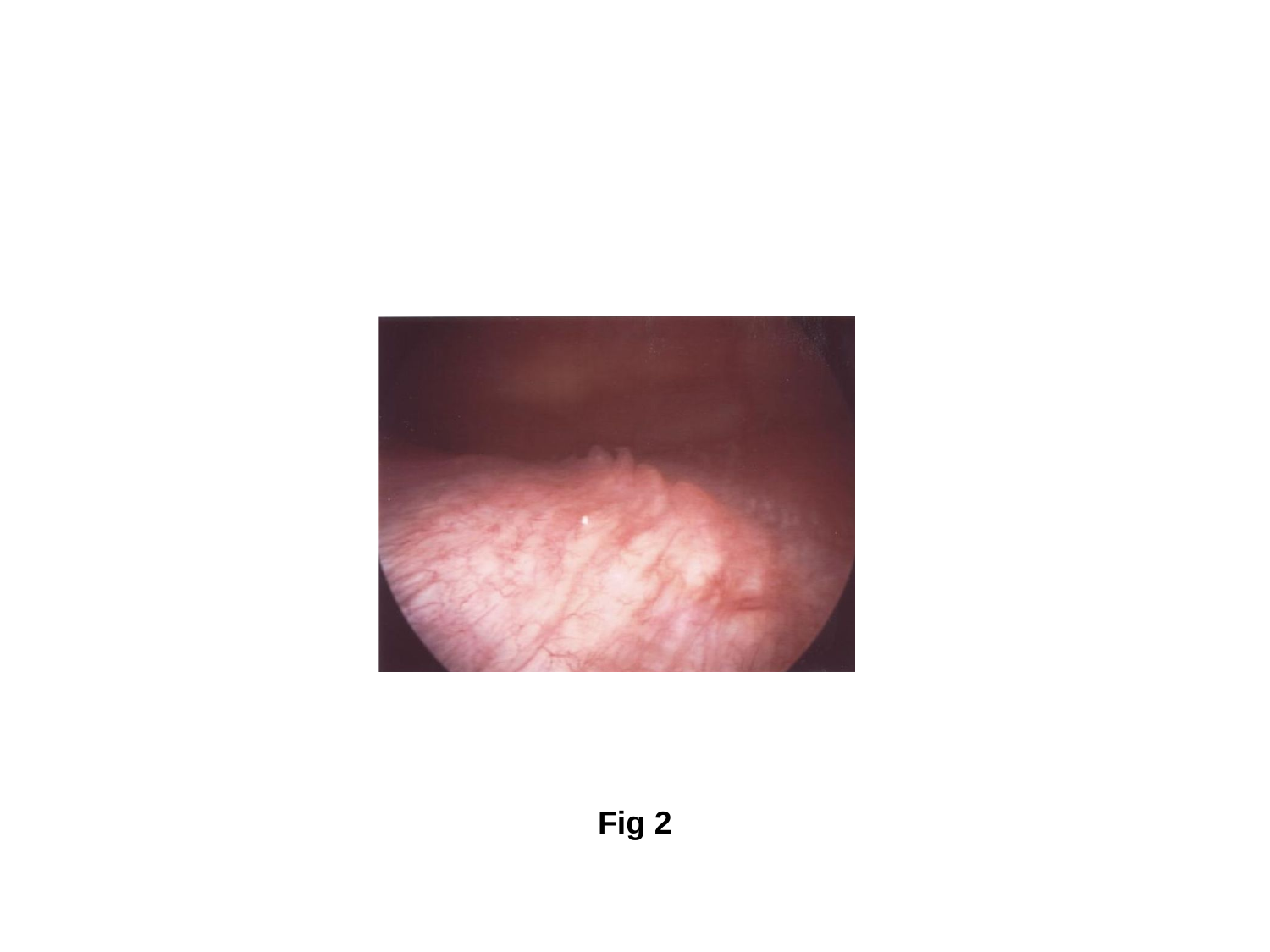

Fig 2

## Slide 4
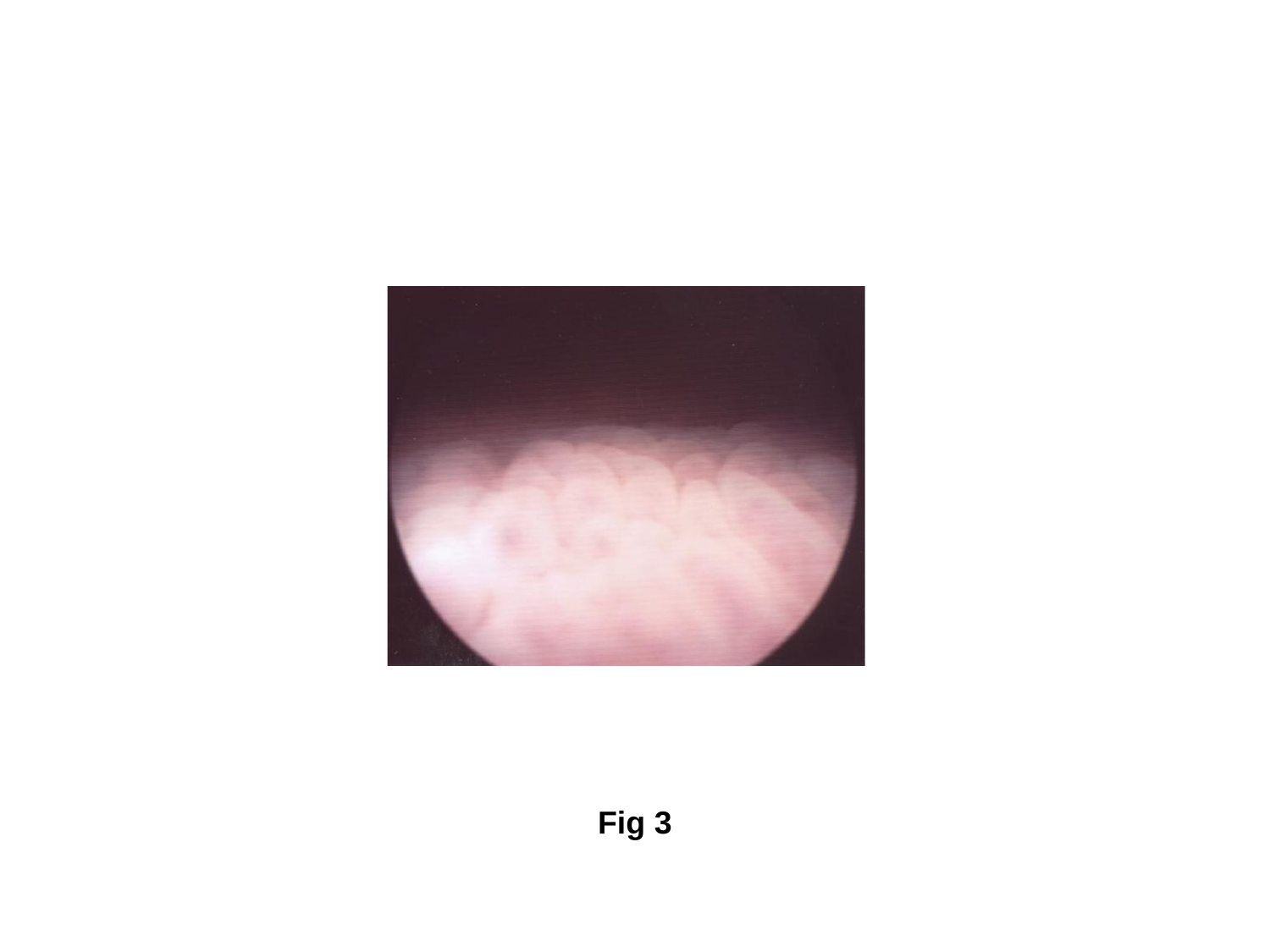

Fig 3

## Slide 5
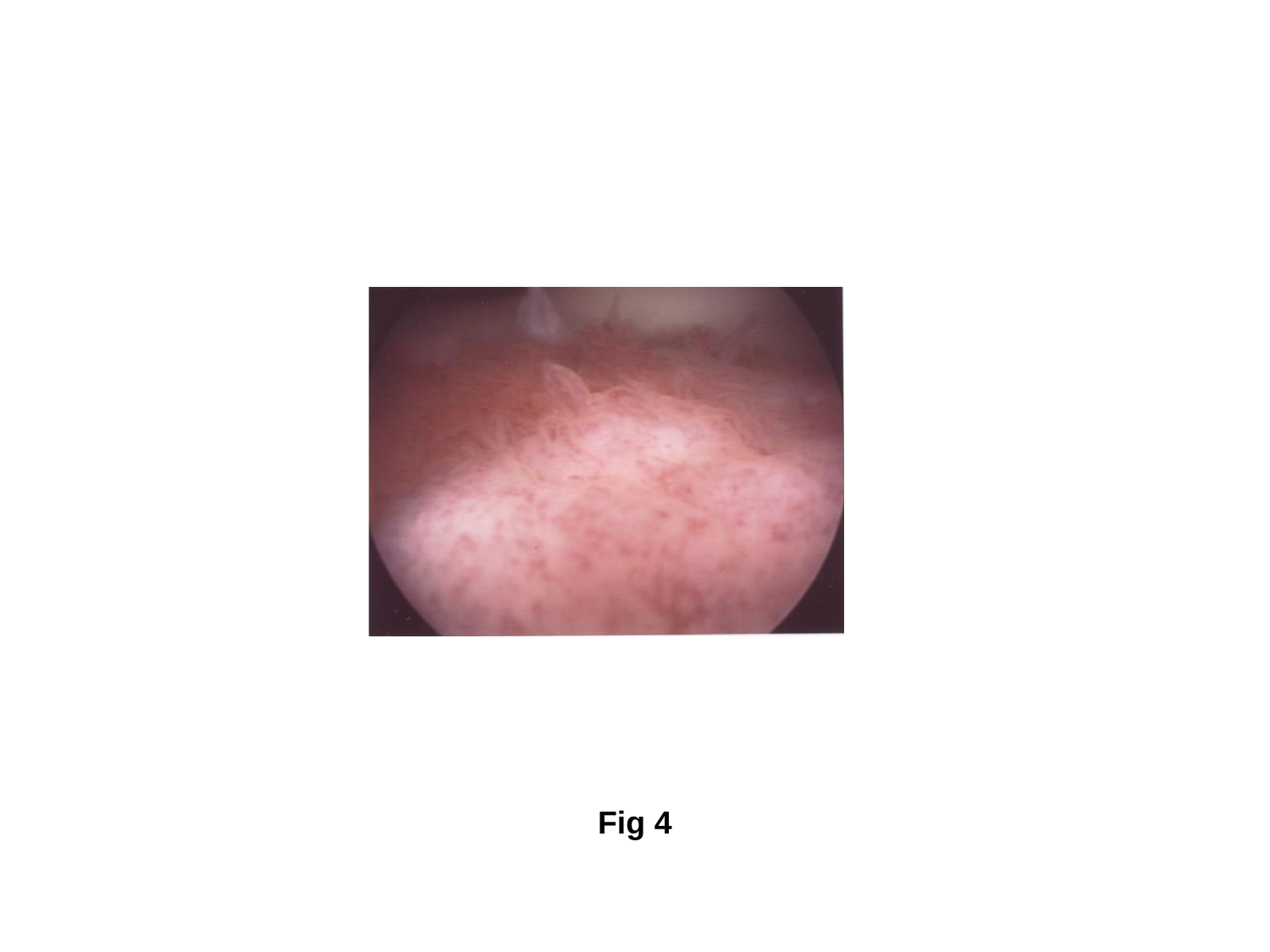

Fig 4

## Slide 6
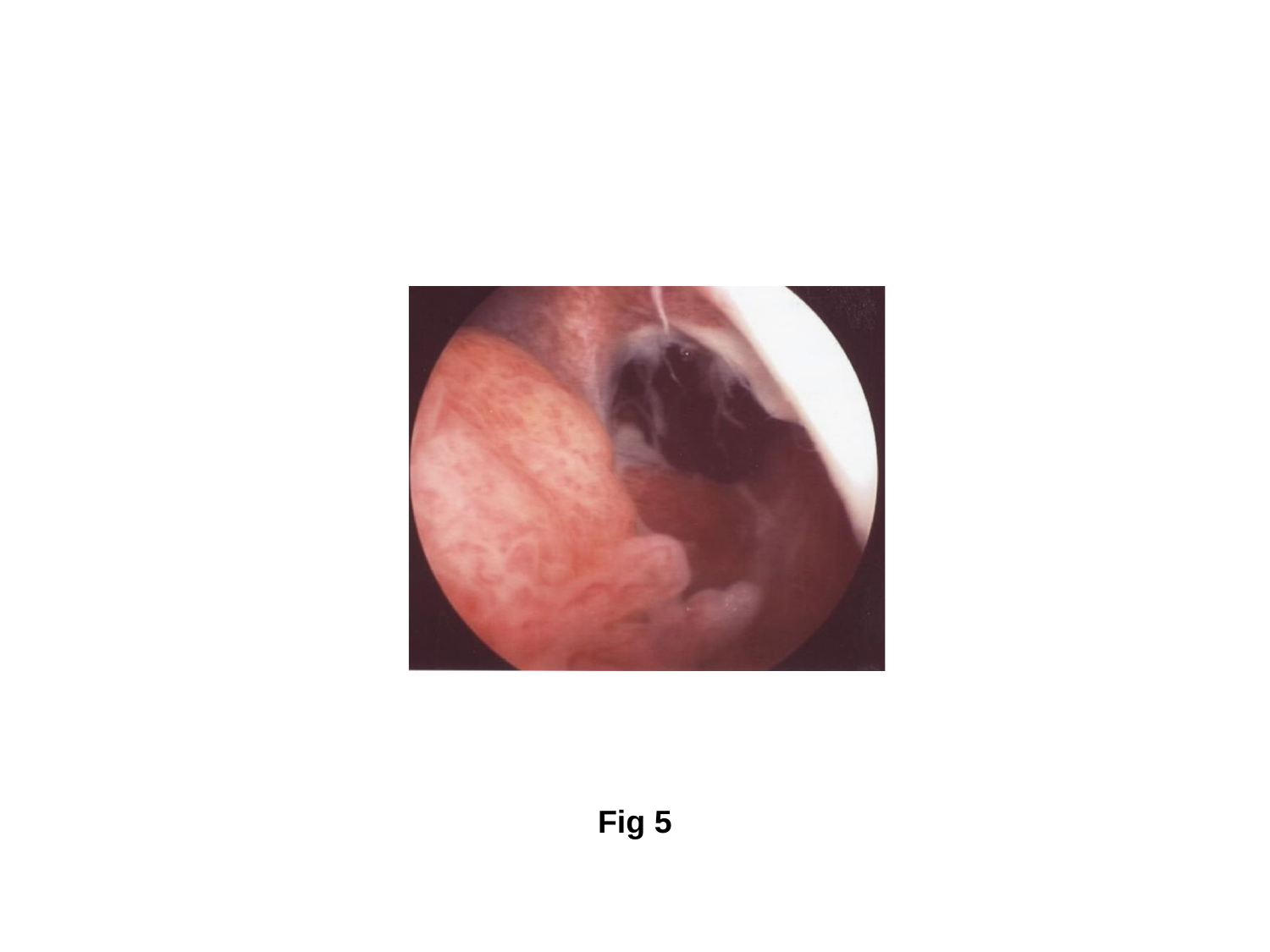

Fig 5

## Slide 7
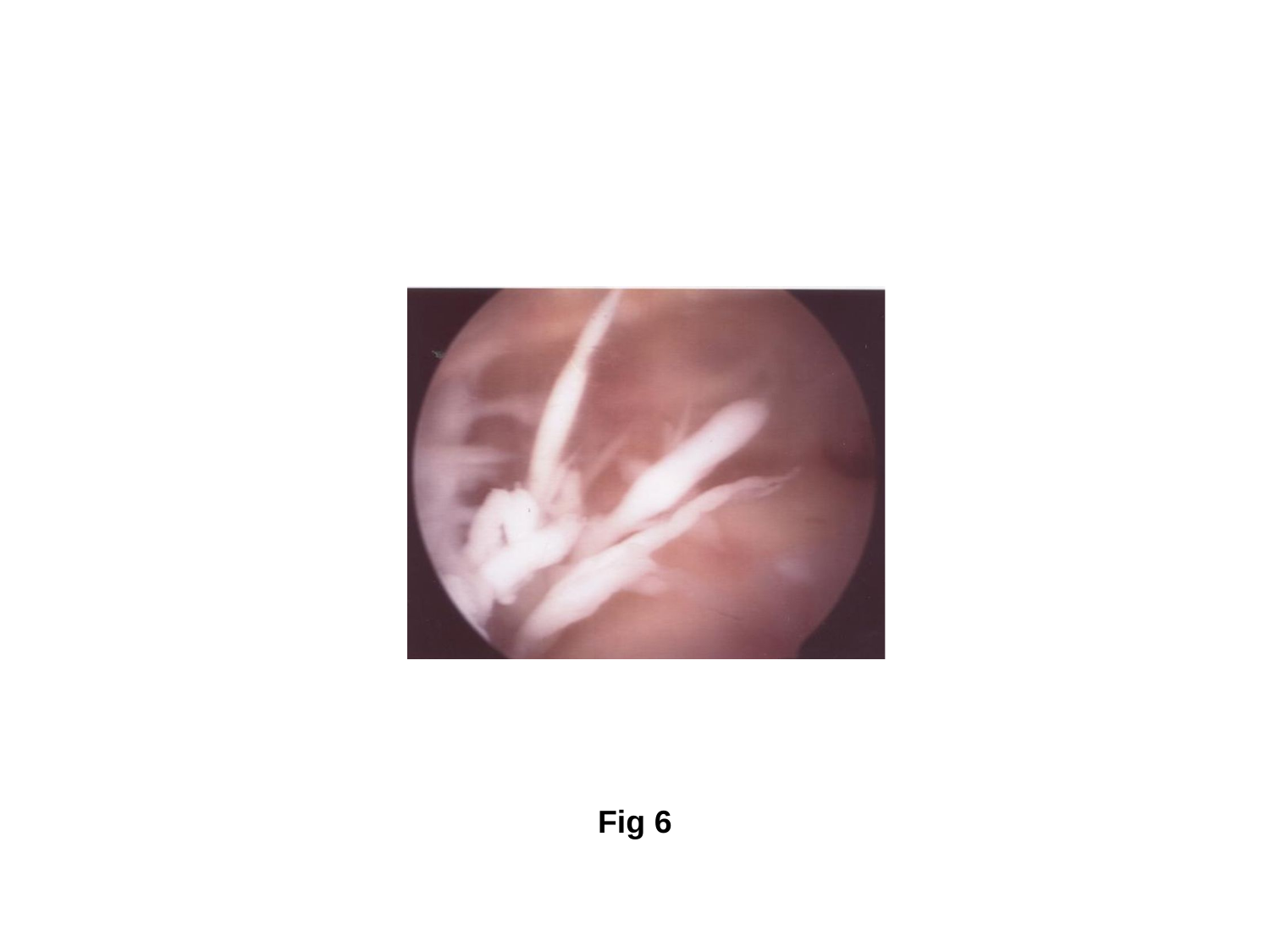

Fig 6

## Slide 8
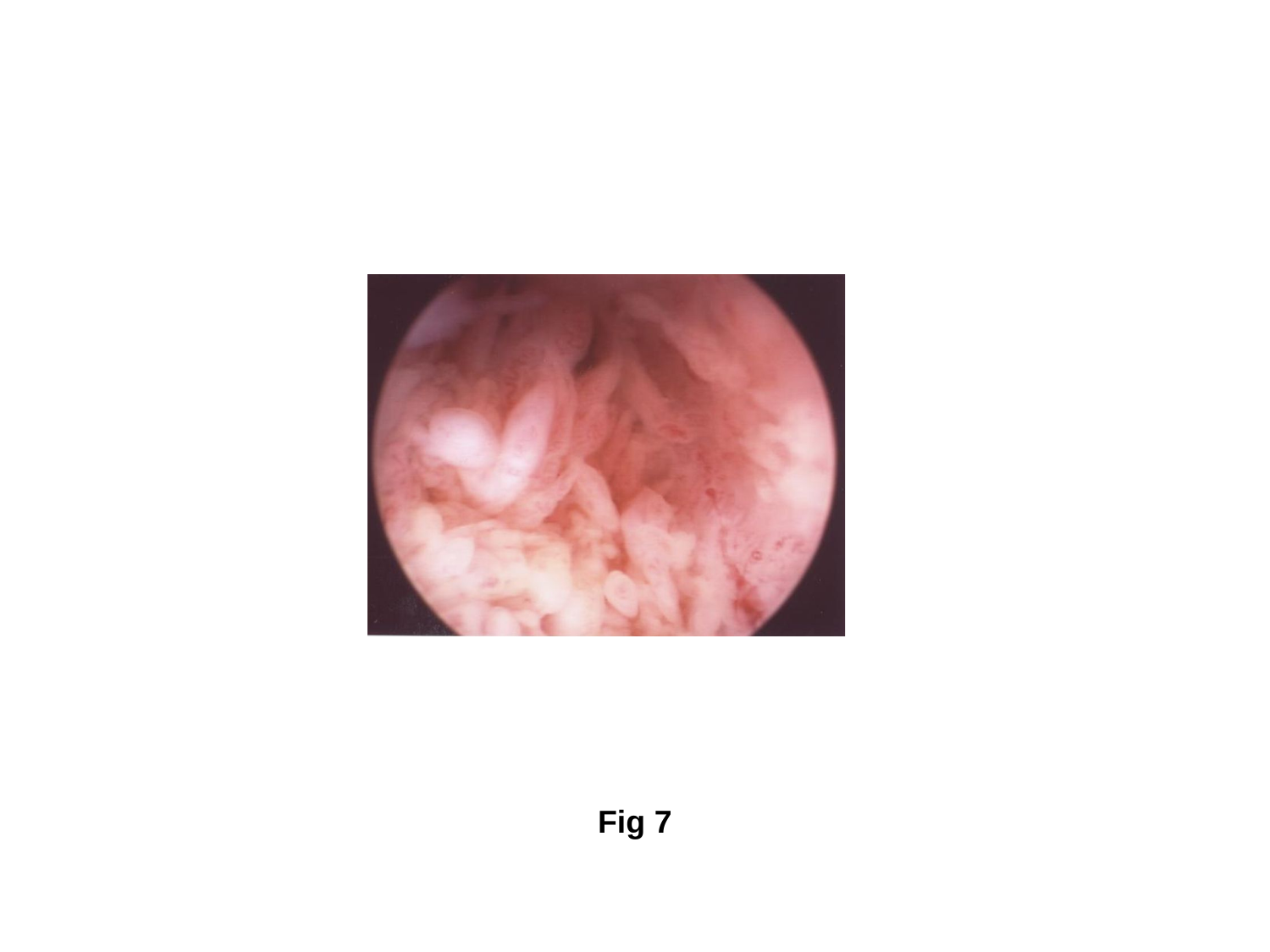

Fig 7

## Slide 9
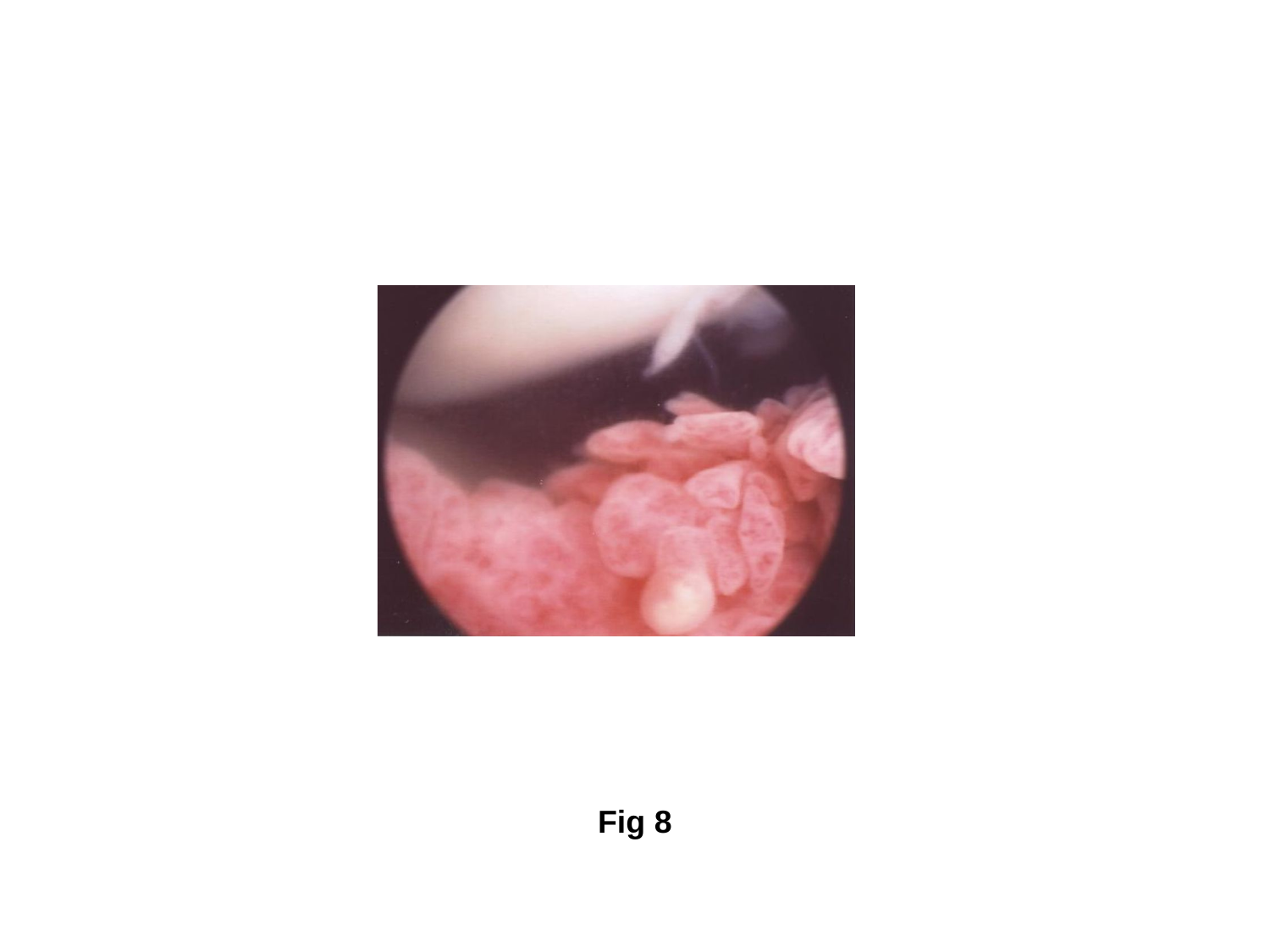

Fig 8

## Slide 10
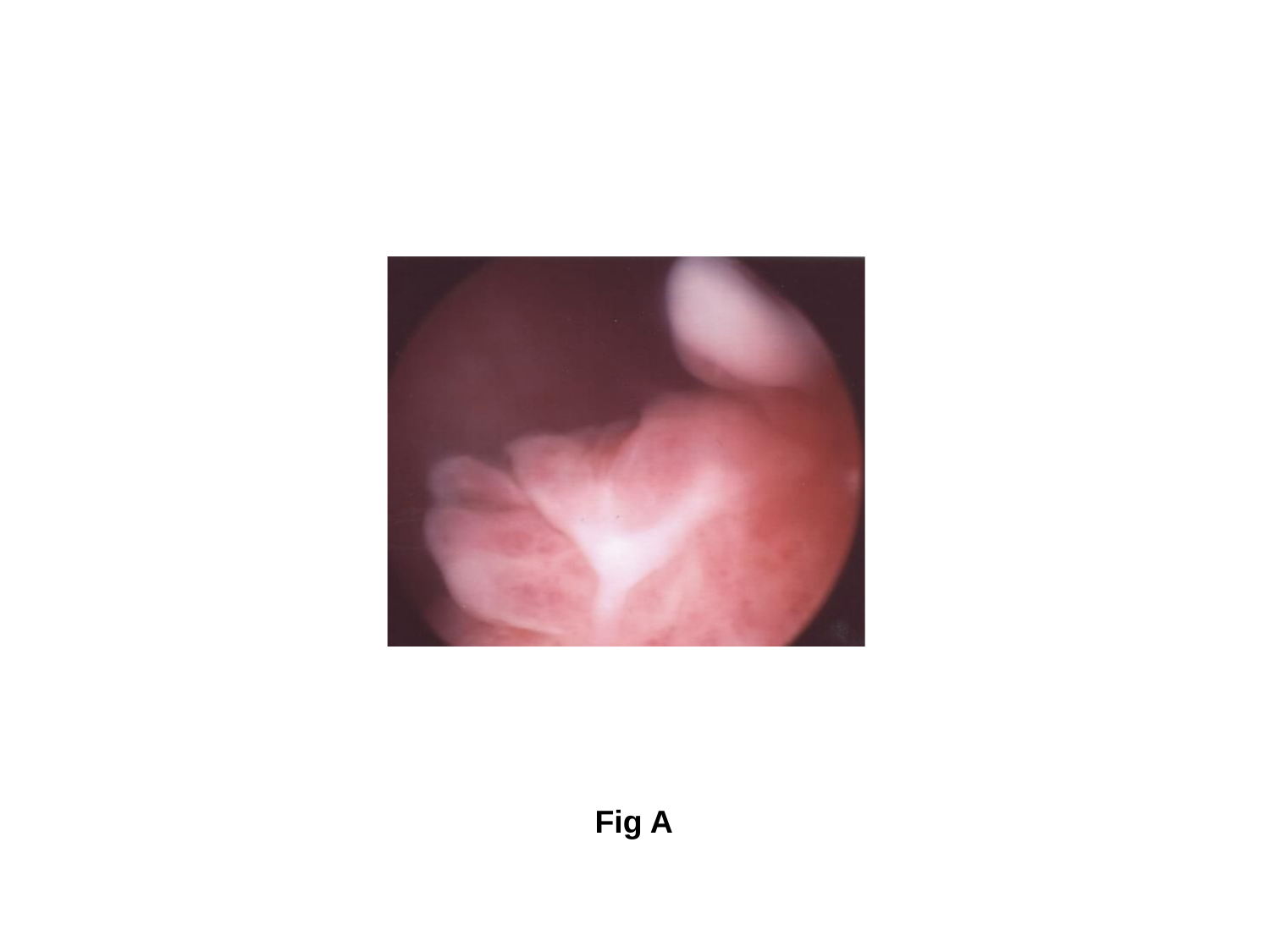

Fig A

## Slide 11
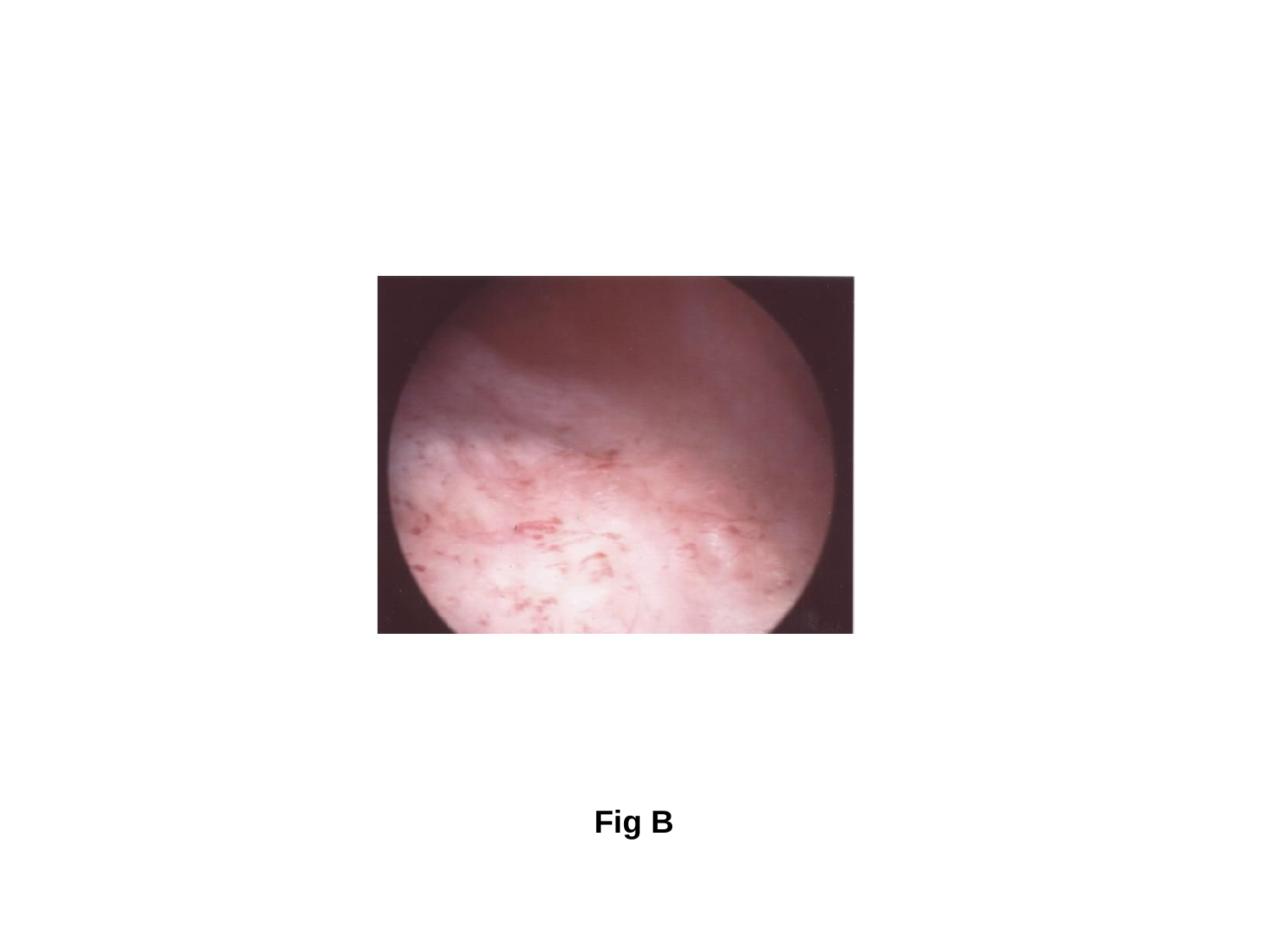

Fig B

## Slide 12
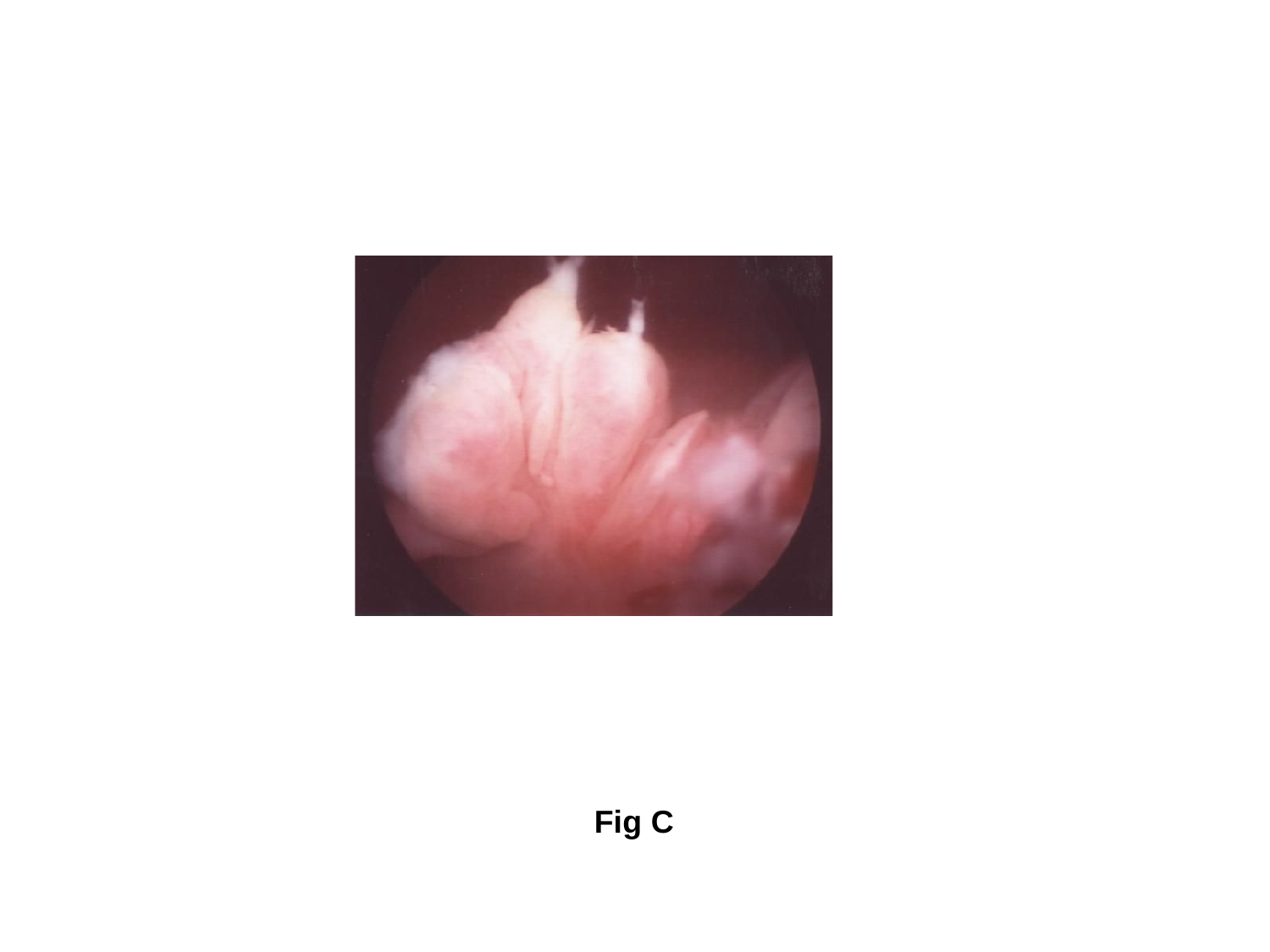

Fig C

## Slide 13
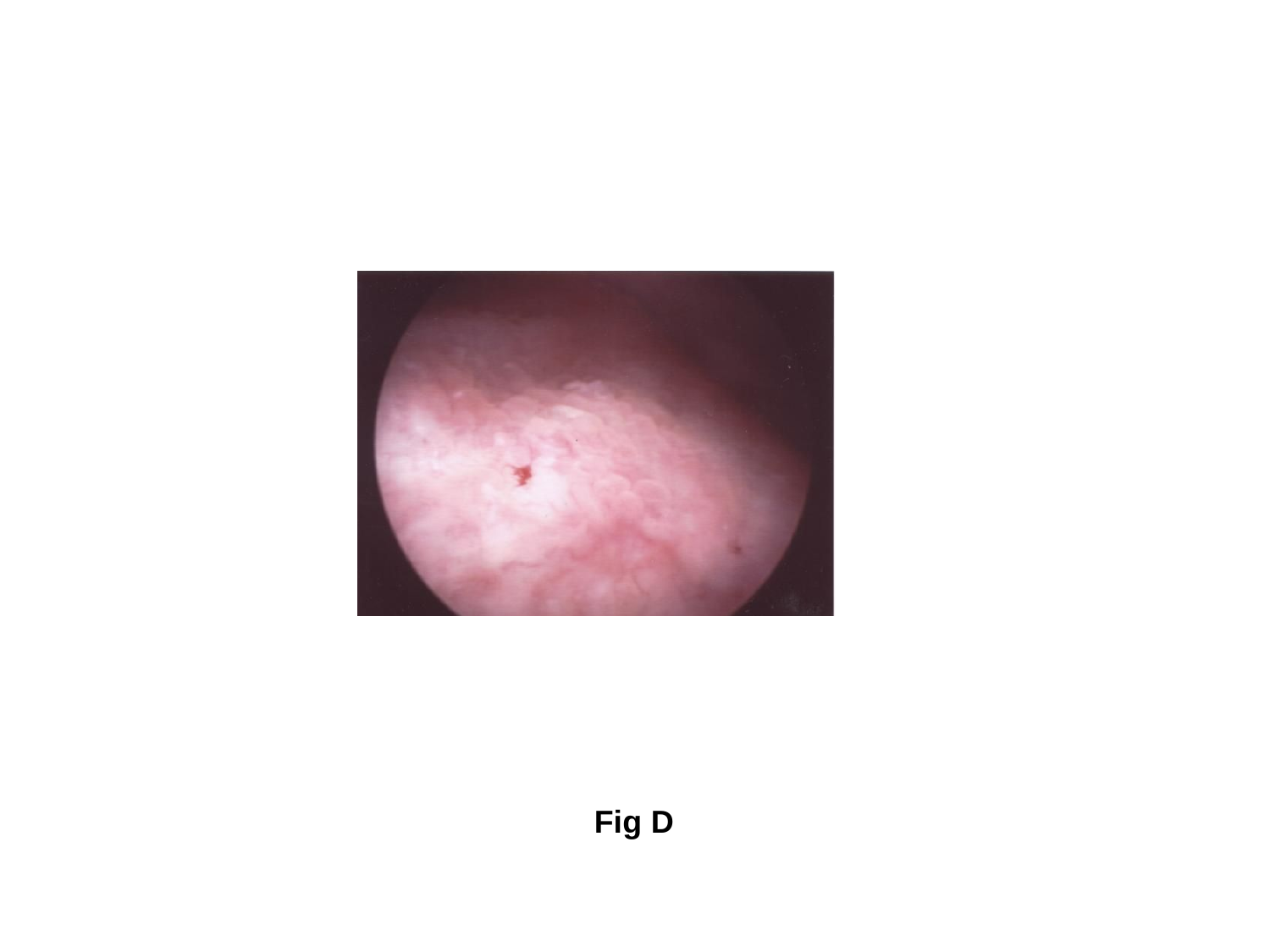

Fig D

## Slide 14
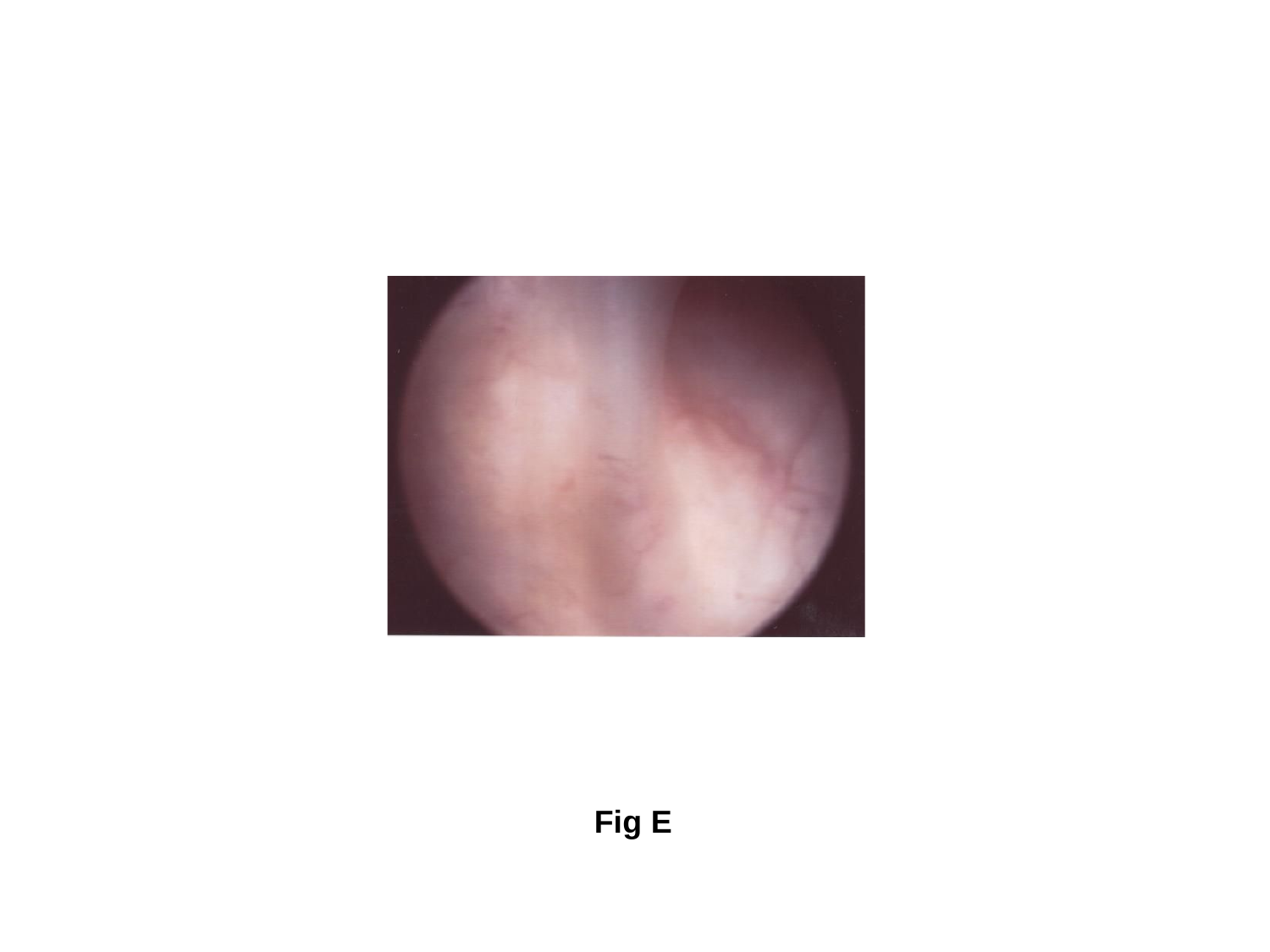

Fig E

## Slide 15
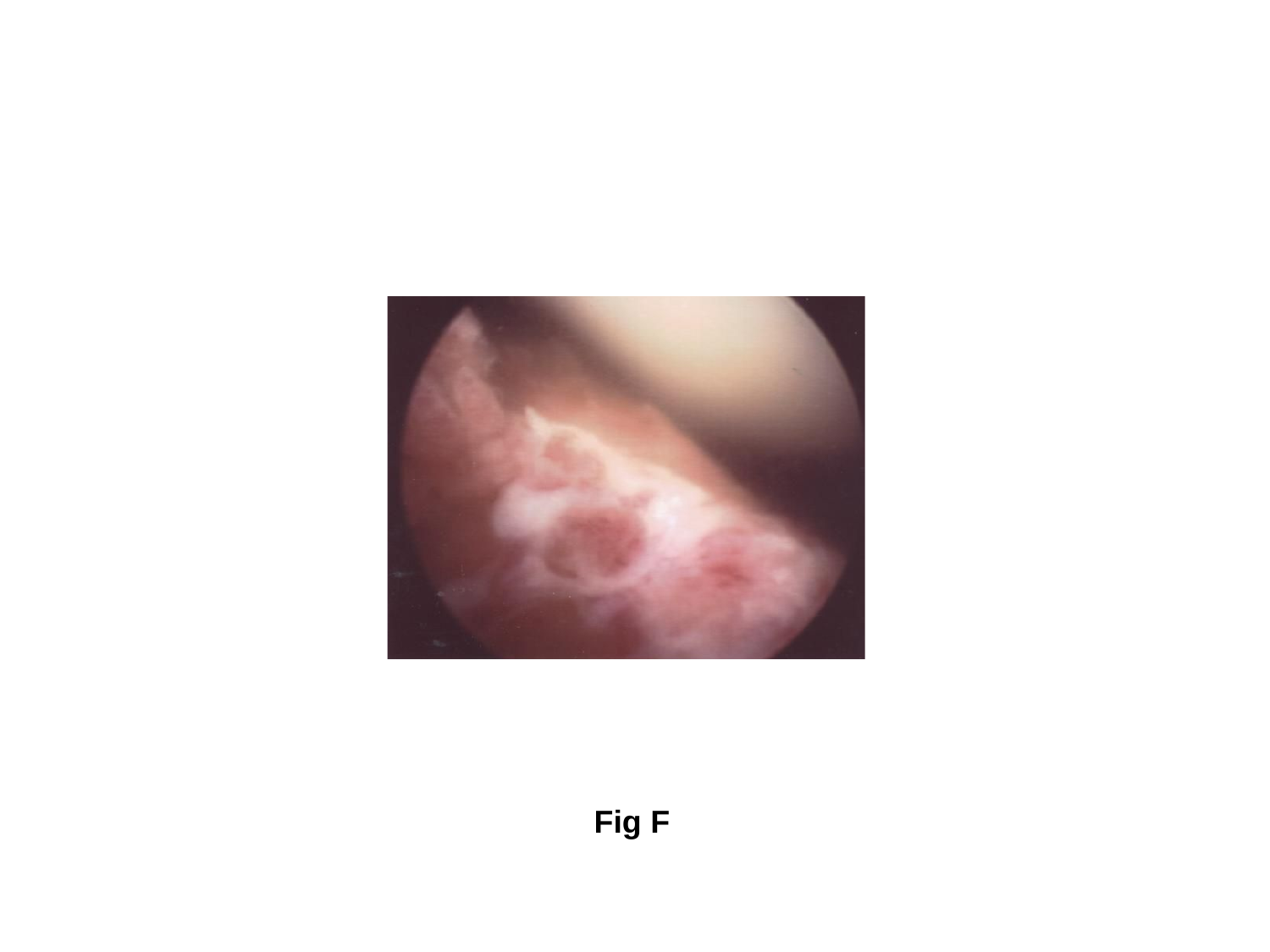

Fig F

## Slide 16
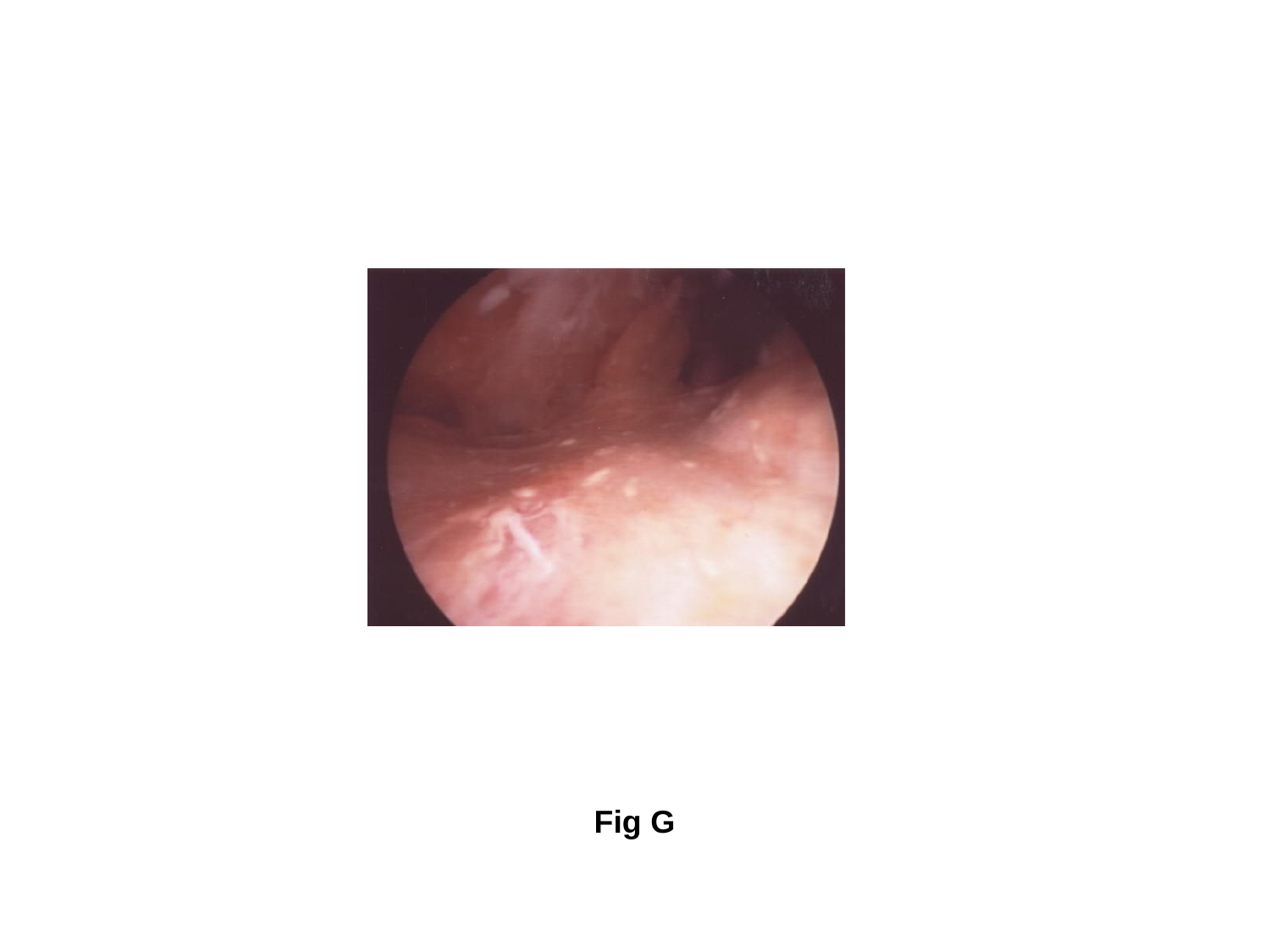

Fig G

## Slide 17
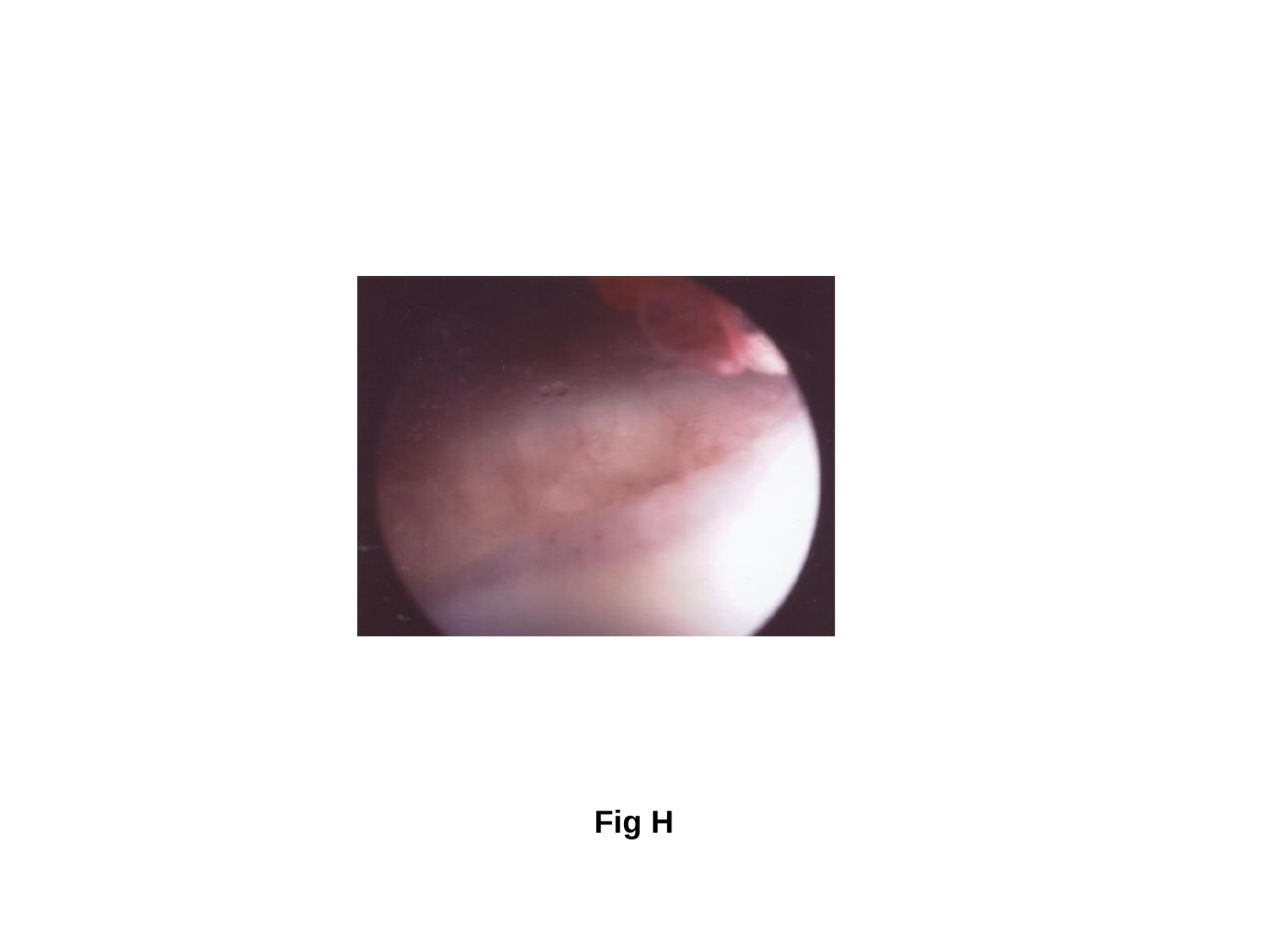

Fig H

## Slide 18
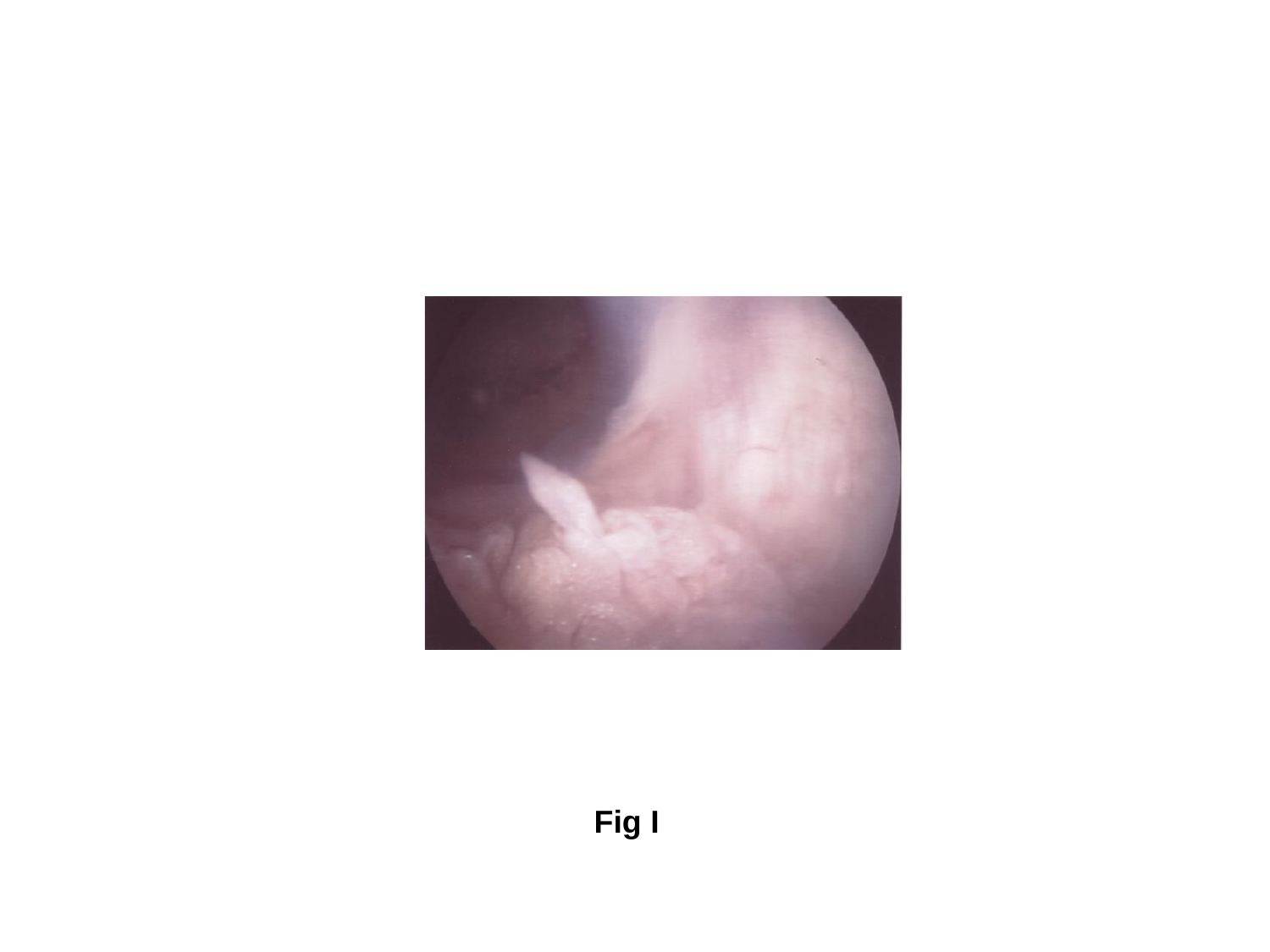

Fig I

## Slide 19
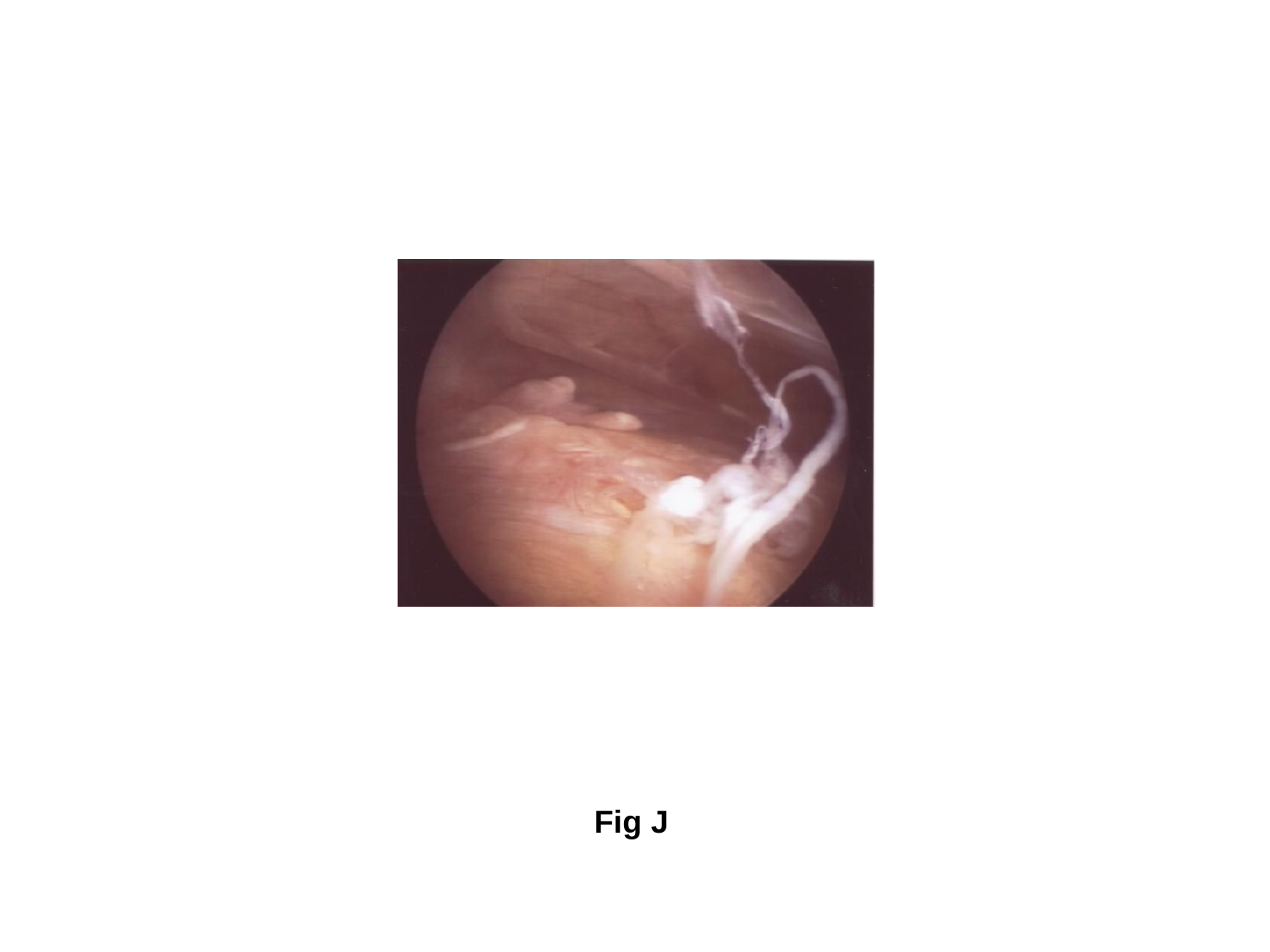

Fig J

## Slide 20
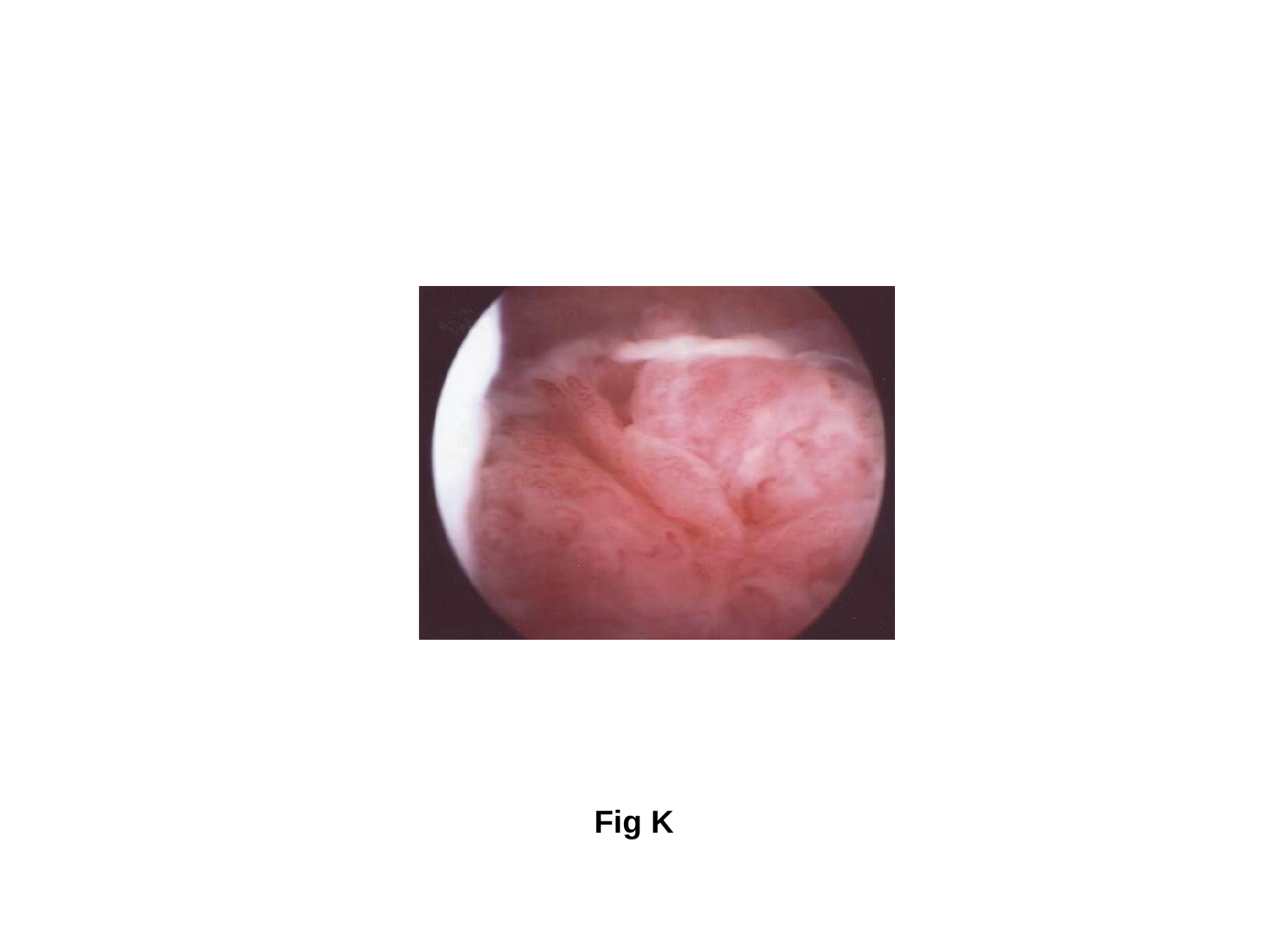

Fig K

## Slide 21
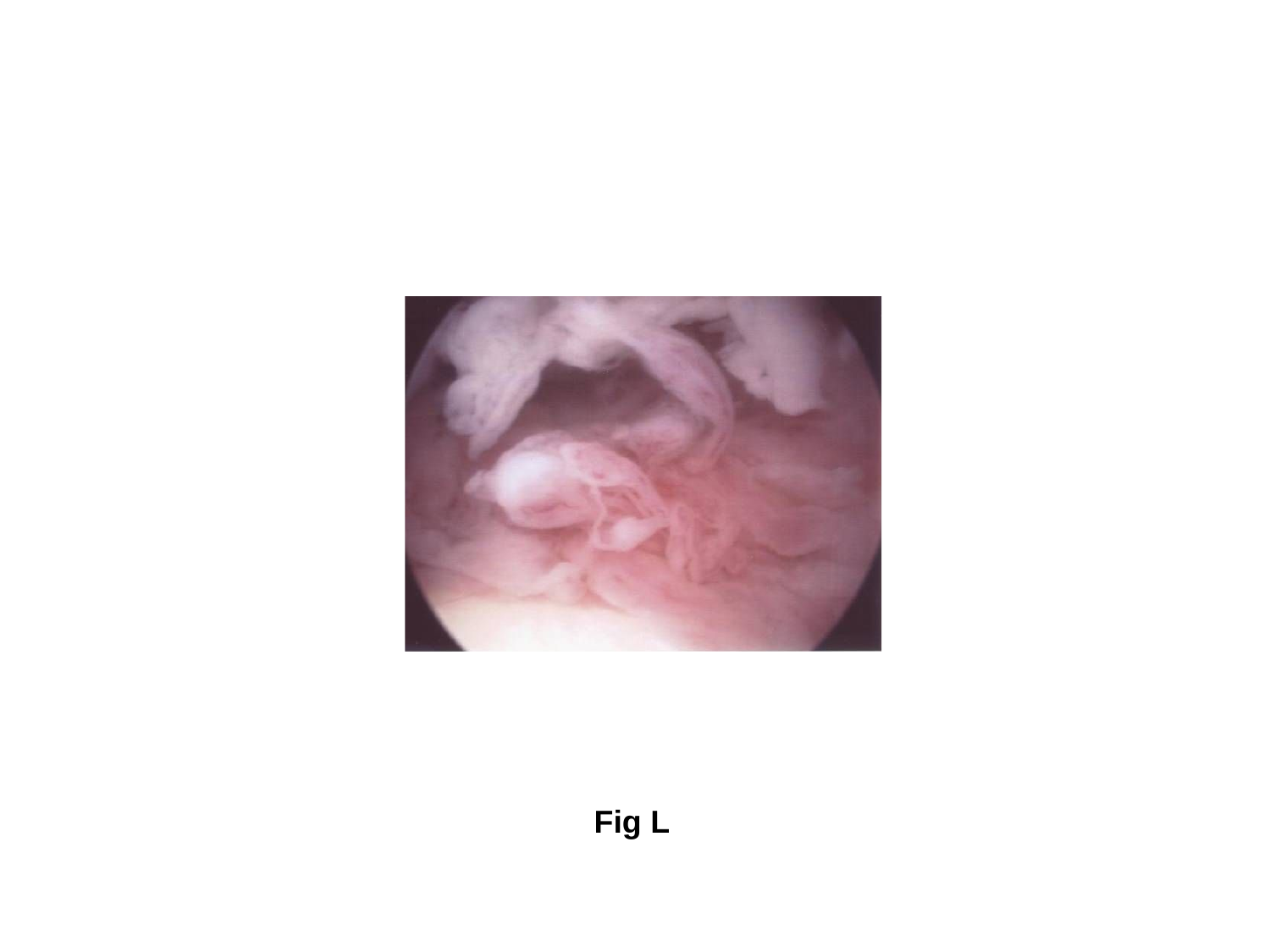

Fig L

## Slide 22
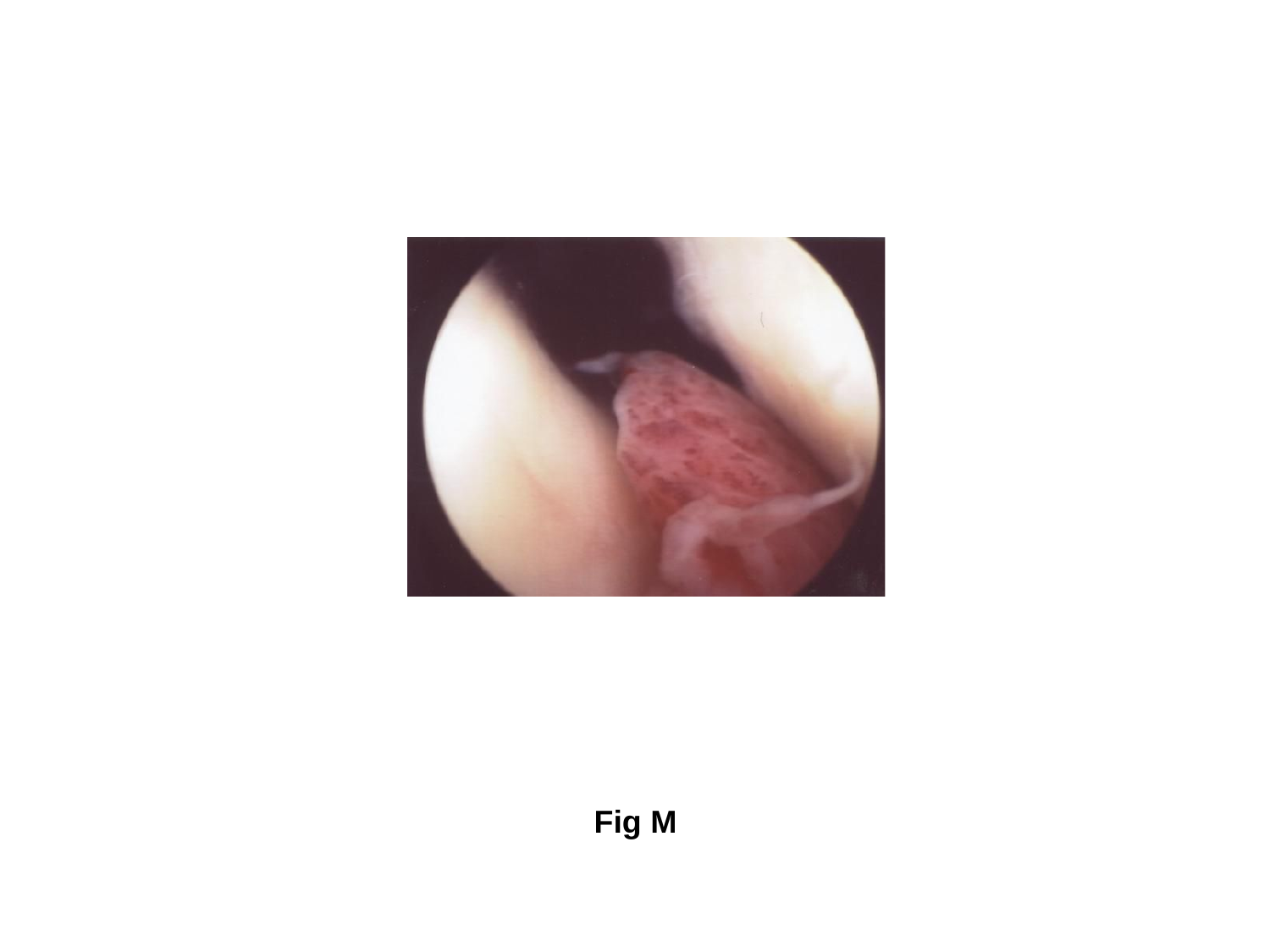

Fig M

## Slide 23
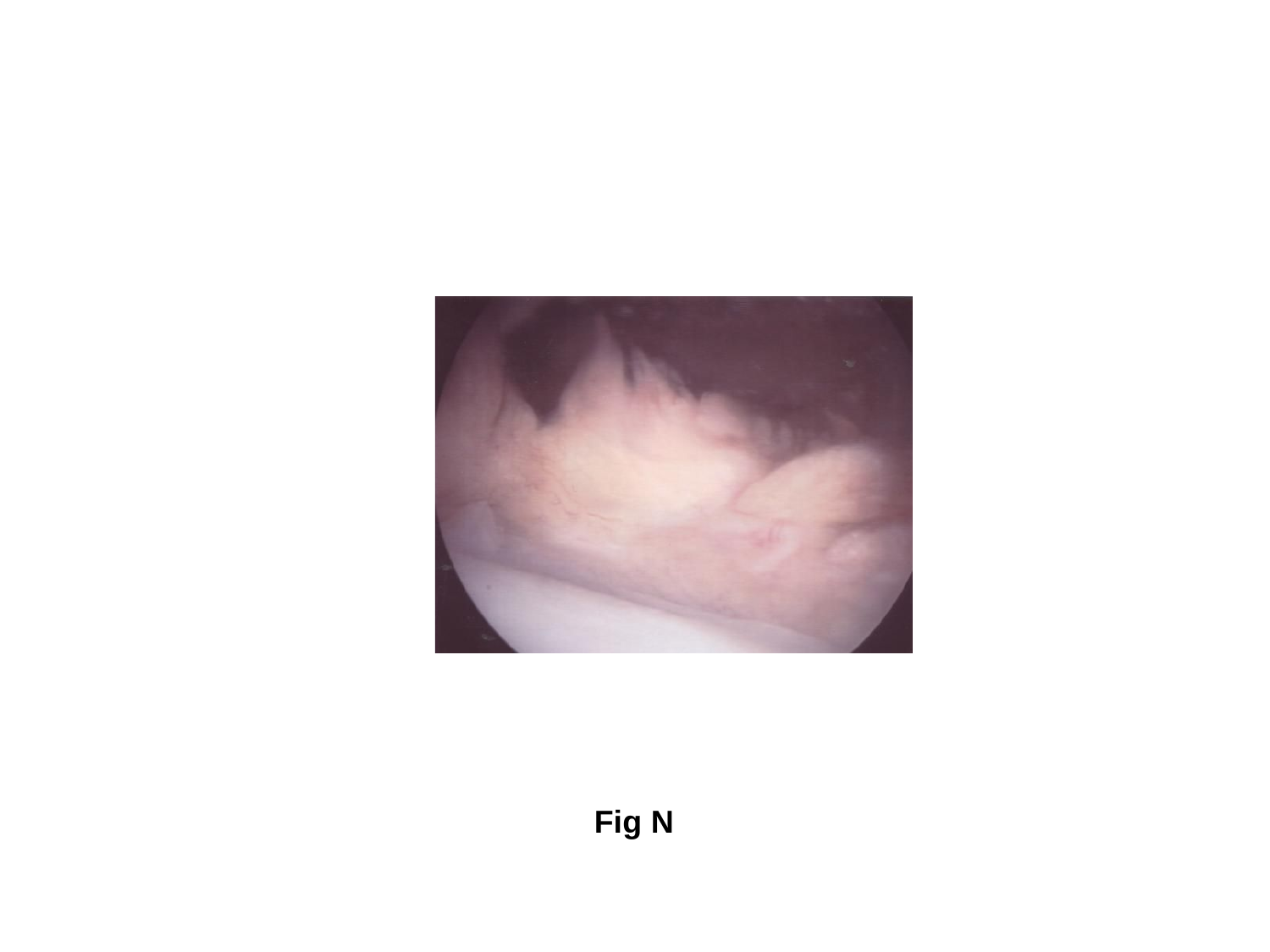

Fig N

## Slide 24
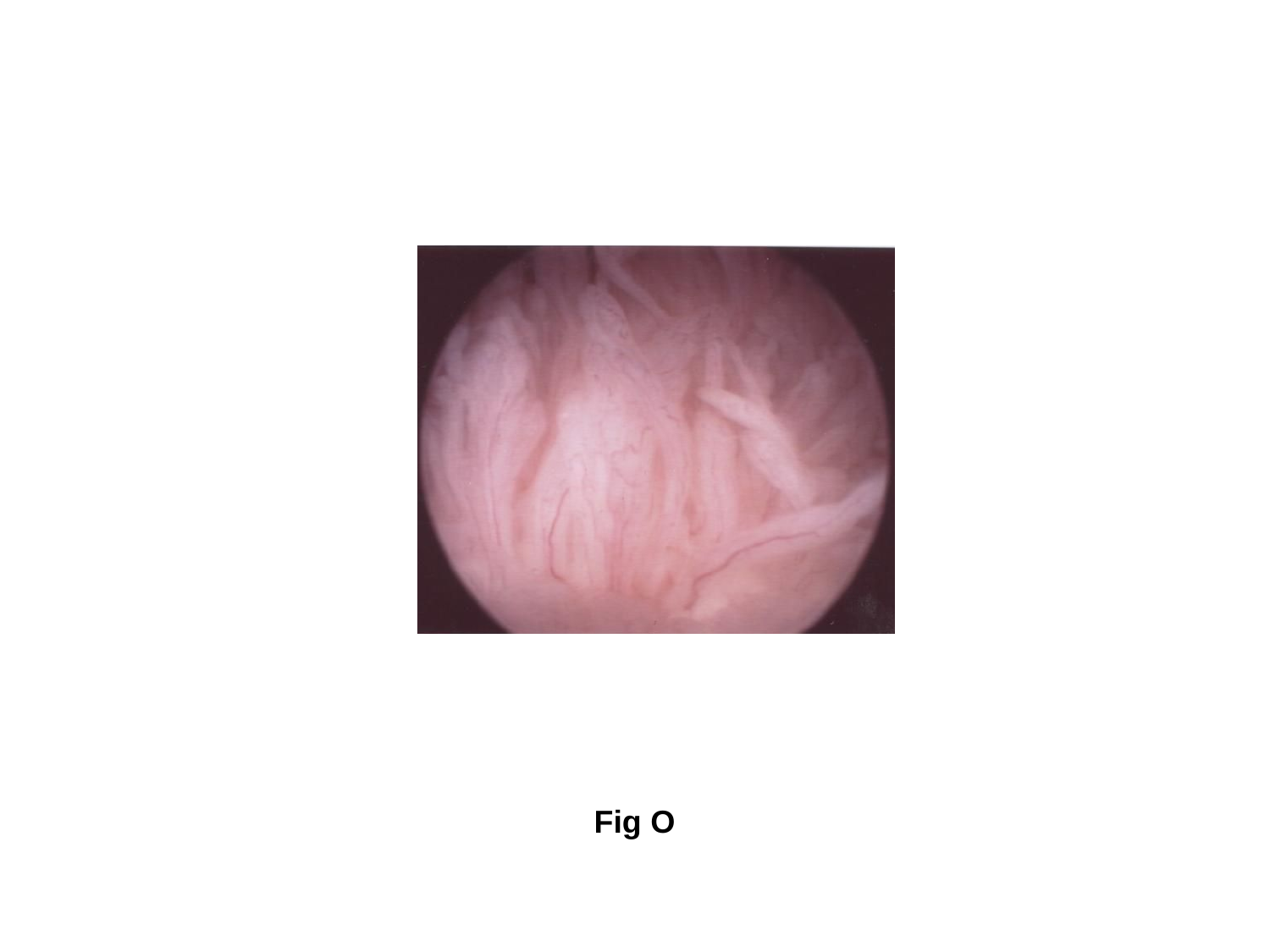

Fig O
